# Supplementary material for: PARP inhibition enhances the antitumor activity of HER3-DXd in non-small cell lung cancer
Source: Cell Rep Med. 2026 Aug 21;7(9):103002. doi: 10.1016/j.xcrm.2026.103002 (PMC13589489; doi:10.1016/j.xcrm.2026.103002)
Supplement: Supplementary file 1 [file mmc1.pdf]

Cell Reports Medicine, Volume *7*

Supplemental information

PARP inhibition enhances the antitumor activity of HER3-DXd in non-small cell lung cancer

Linh Lin, Narges Moradi, Iris A. Lähdeniemi, Hanna K. Laitinen, Bassel Alsaed, Lilja Lahtinen, Timothy Lopez, Shadi Jansouz, Aine Knott, Kara M. Soroko, Ilkka Ilonen, Prafulla C. Gokhale, Pasi A. Jänne, and Heidi M. Haikala

Supplemental PDF (Linh et al., Cell Reports Medicine)

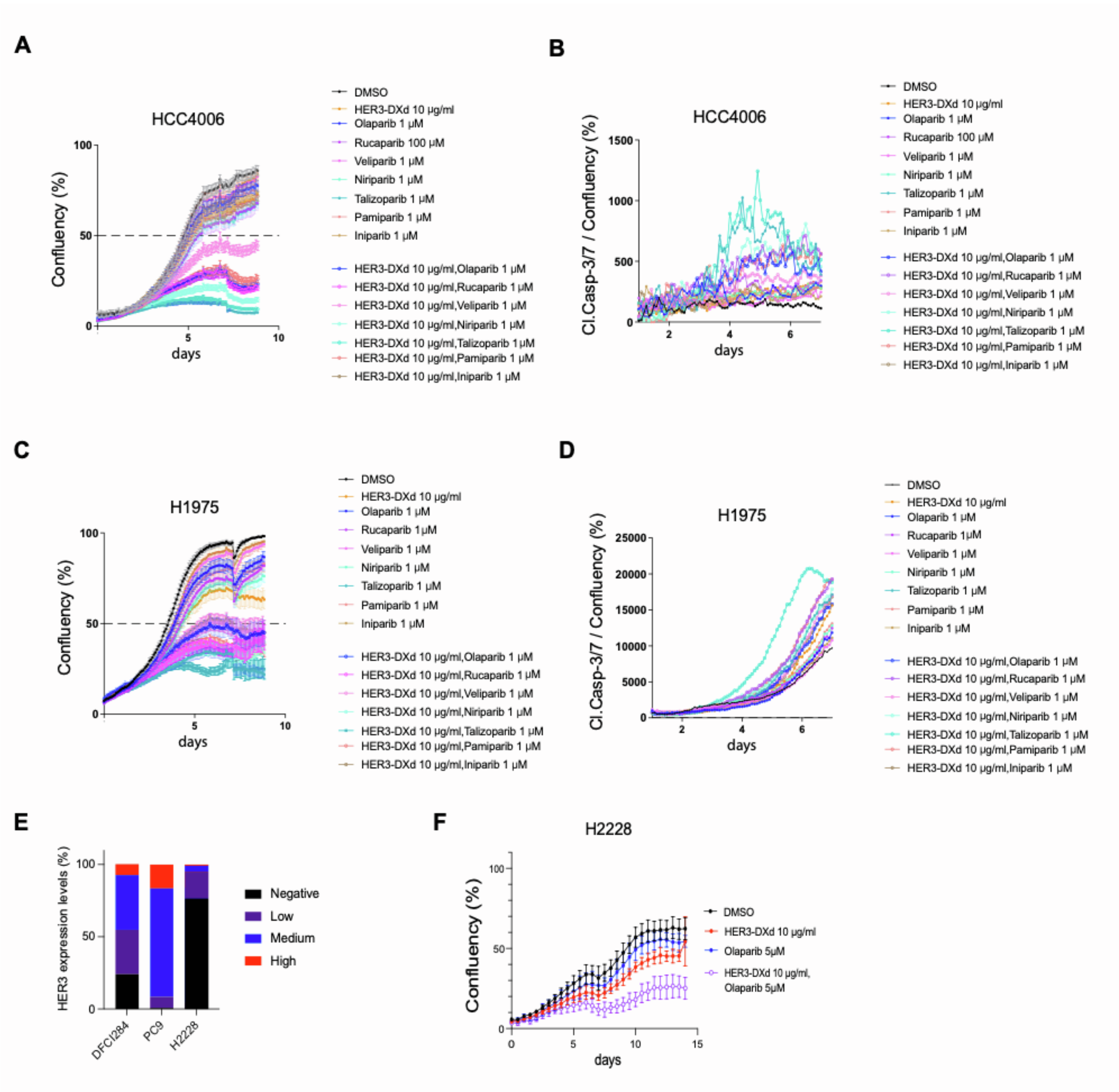

Figure S1: Additional data from EGFR-mutant cell lines (related to Figure 2). (A-D) Incucyte cell confluency and apoptosis assays from PARP inhibitor and HER3-DXd-treated cells over time. Data are representative of three individual experiments. (E) Flow cytometry quantification of HER-3 expression levels in DFCI284, PC9 and H2228, n = 3 biological experiments. (F) CellCyte cell confluency assay of H2228 treated with PARP inhibitor and HER3-DXd over time. Data are presented as mean ± SD. Data are representative of three individual experiments.

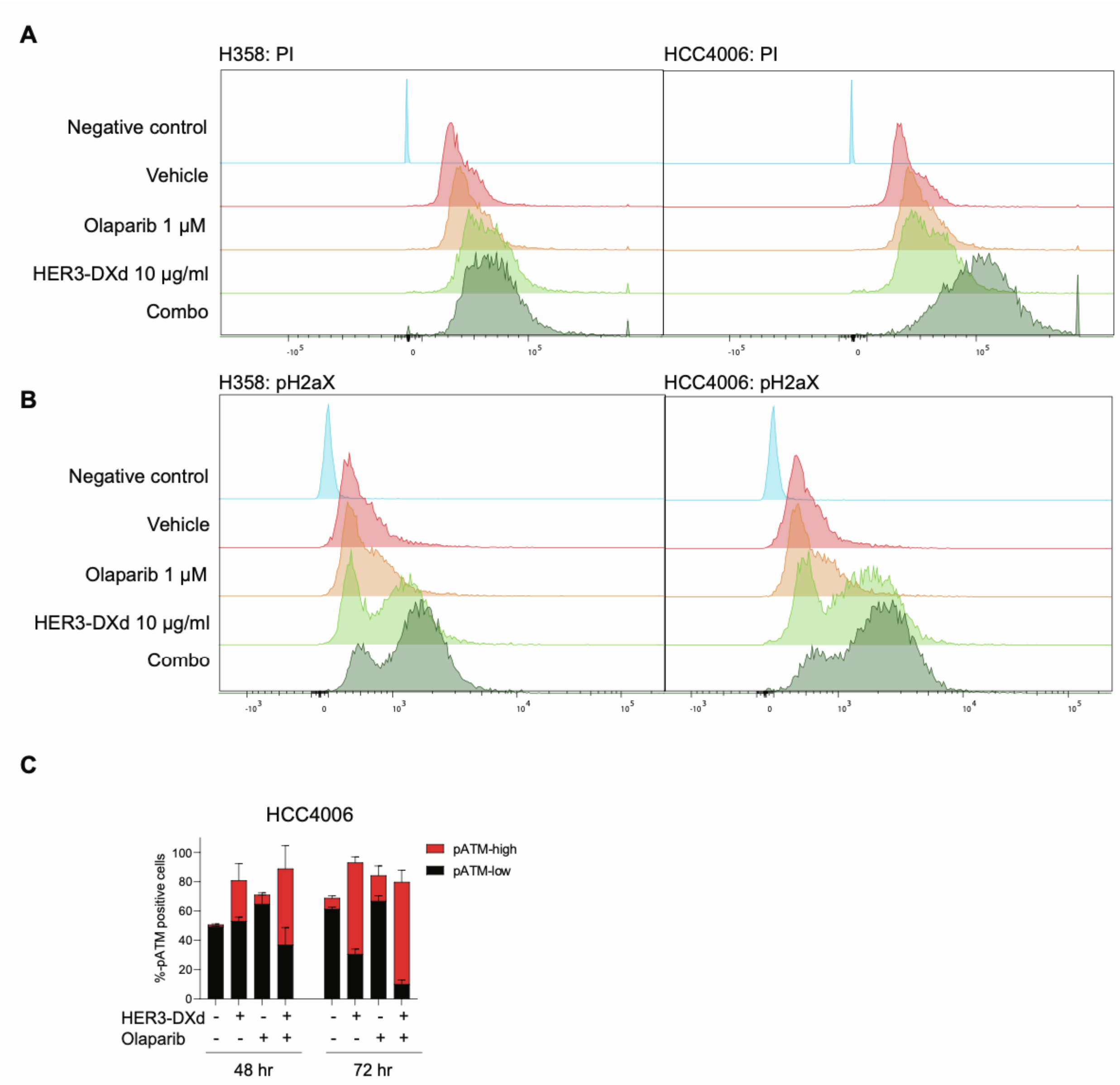

Figure S2: DNA damage response activation in H358 and HCC4006 (related to Figure 4). (A-B) Representative flow cytometry plots of PI and pH2aX staining in H358 and HCC4006 cells. (C) Quantification of pATM flow cytometry staining in HCC4006 cell line, n = 3 biological experiments. Data are presented as mean ± SD.

A

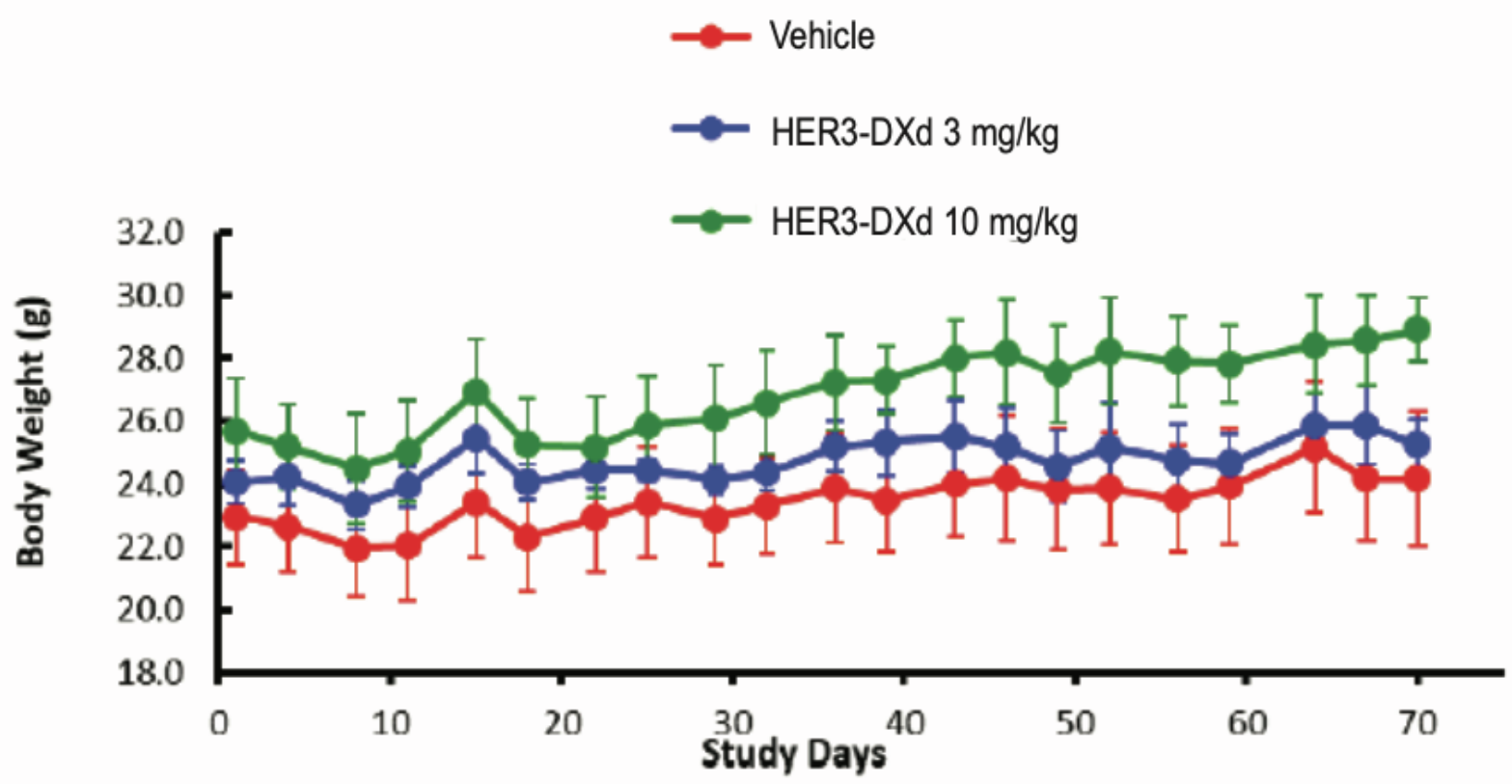

B

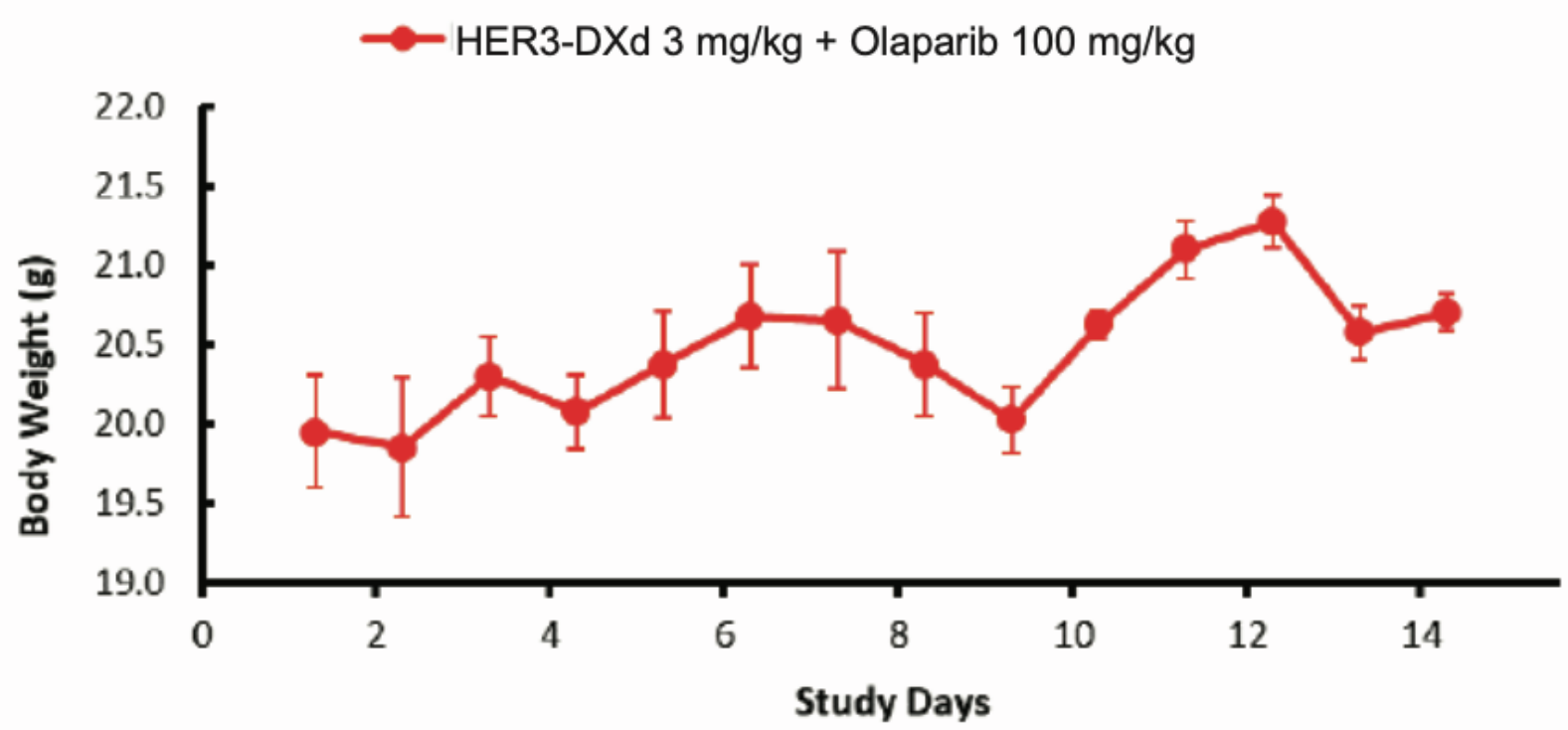

C

Pharmacodynamics Study Groups					
Group #	Test Agent	Dose	Dosing Volume	Dosing Schedule	Number of Animals
1	Vehicle	-	10 mL/kg	PO QD x 3 Days	3
2	HER3-DXd	3 mg/kg	10 mL/kg	IV x 1 Dose	3
3	Olaparib	100 mg/kg	10 mL/kg	PO QD x 3 Days	3
4	HER3-DXd + Olaparib	3 mg/kg + 100 mg/kg	10 mL/kg + 10 mL/kg	IV x 1 Dose + PO QD x 3 Days	3

Figure S3: *In vivo* dose optimization and tolerability data in mice (related to Figure 5). (A) Average body weight plot for pilot efficacy study represented as mean ± SEM. (B) Tolerability study with four female NSG mice weighed daily for two weeks represented as mean ± SEM. (C) Pharmacodynamics studies for the optimization of the drug dose and dosing strategies in mice.

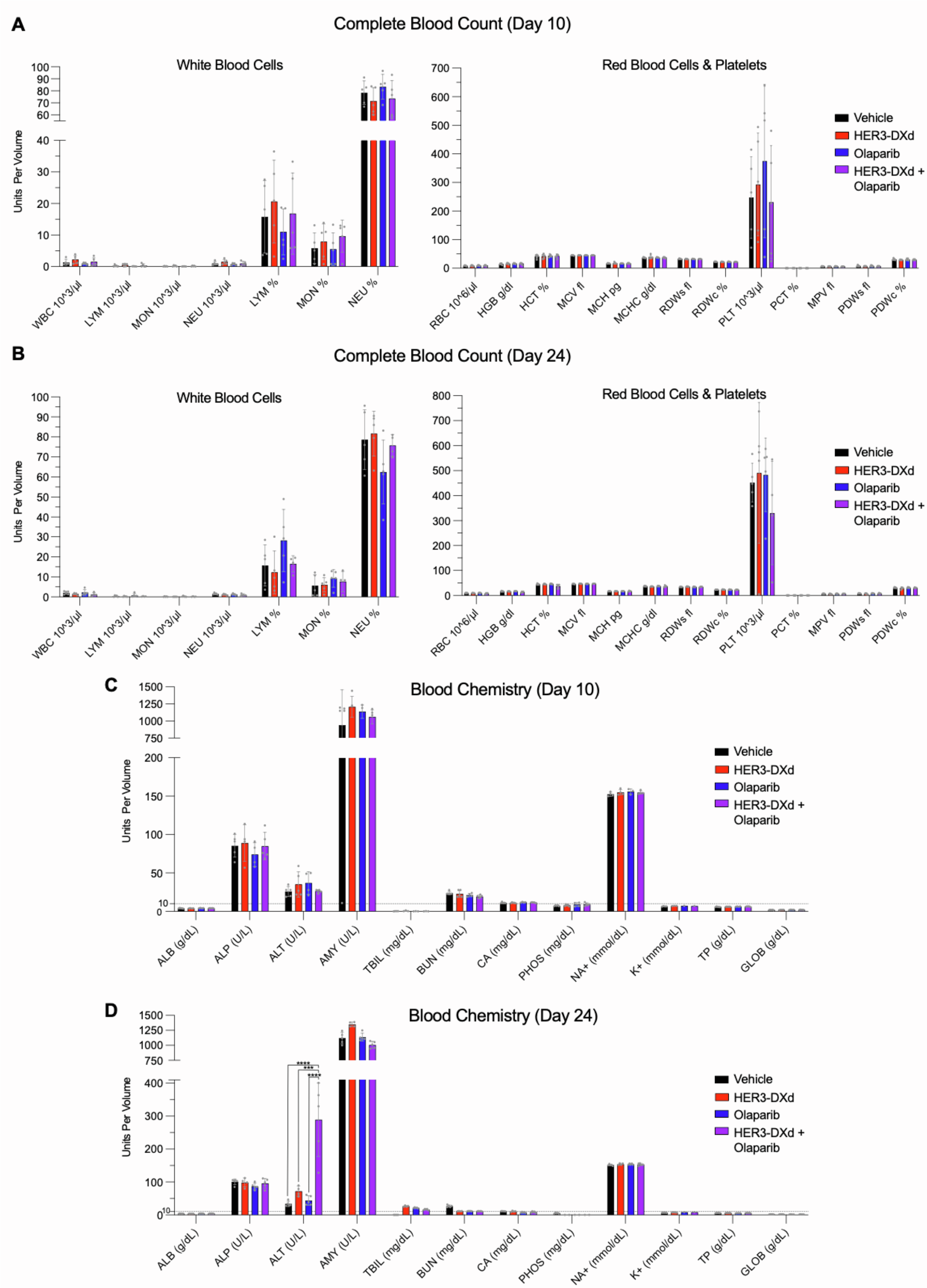

A
Complete Blood Count (Day 10)
White Blood Cells
Red Blood Cells & Platelets
Units Per Volume
WBC 10^3/µl
LYM 10^3/µl
MON 10^3/µl
NEU 10^3/µl
LYM %
MON %
NEU %
RBC 10^6/µl
HGB g/dl
HCT %
MCV fl
MCH pg
MCHC g/dl
RDWs fl
RDWc %
PLT 10^3/µl
PCT %
MPV fl
PDWs fl
PDWc %
Vehicle
HER3-DXd
Olaparib
HER3-DXd + Olaparib
B
Complete Blood Count (Day 24)
White Blood Cells
Red Blood Cells & Platelets
C
Blood Chemistry (Day 10)
ALB (g/dL)
ALP (U/L)
ALT (U/L)
AMY (U/L)
TBIL (mg/dL)
BUN (mg/dL)
CA (mg/dL)
PHOS (mg/dL)
NA+ (mmol/dL)
K+ (mmol/dL)
TP (g/dL)
GLOB (g/dL)
D
Blood Chemistry (Day 24)
****
***
****

Figure S4: ***In vivo*** **tolerability data of HER3-DXd in mice (related to Figure 5).**

(A) Complete blood count of mice on day 10. (B) Complete blood count of mice on day 24. (C) Blood chemistry of mice on day 10. (D) Blood chemistry of mice on day 24. Data are presented as mean ± SD, n = 5 mice per condition. One-way ANOVA. Treatments included HER3-DXd (3 mg/kg), olaparib (100 mg/kg), or their combination. WBC, white blood cells; NEU, neutrophils; LYM, lymphocytes; MON, monocytes; EOS, eosinophils; BAS, basophils; IMG, immature granulocytes; NEU%, percentage of neutrophils; LYM%, percentage of lymphocytes; MON%, percentage of monocytes; EOS%, percentage of eosinophils; BAS%, percentage of basophils; IMG%, percentage of immature granulocytes; RBC, red blood cells; HGB, hemoglobin; HCT, hematocrit; MCV, mean corpuscular volume; MCH, mean corpuscular hemoglobin; MCHC, mean corpuscular hemoglobin concentration; RDW-CV, coefficient of variation in red cell distribution width; RDW-SD, standard deviation in red cell distribution width; PLT, platelets; MPV, mean platelet volume; PDW, platelet distribution width; PCT, procalcitonin; P-LCC, platelet larger cell count; P-LCR, platelet larger cell ratio. ALB, albumin; ALP, alkaline phosphatase; ALT, alanine transaminase; AMY, amylase; TBIL, total bilirubin; PHOS, phosphorus; GLU, glucose; TP, total protein; GLOB, globulin.

Unless otherwise specified, results were not significant.

P < 0.05; ** P < 0.01; *** P < 0.001; **** P < 0.0001; ns, not significant.

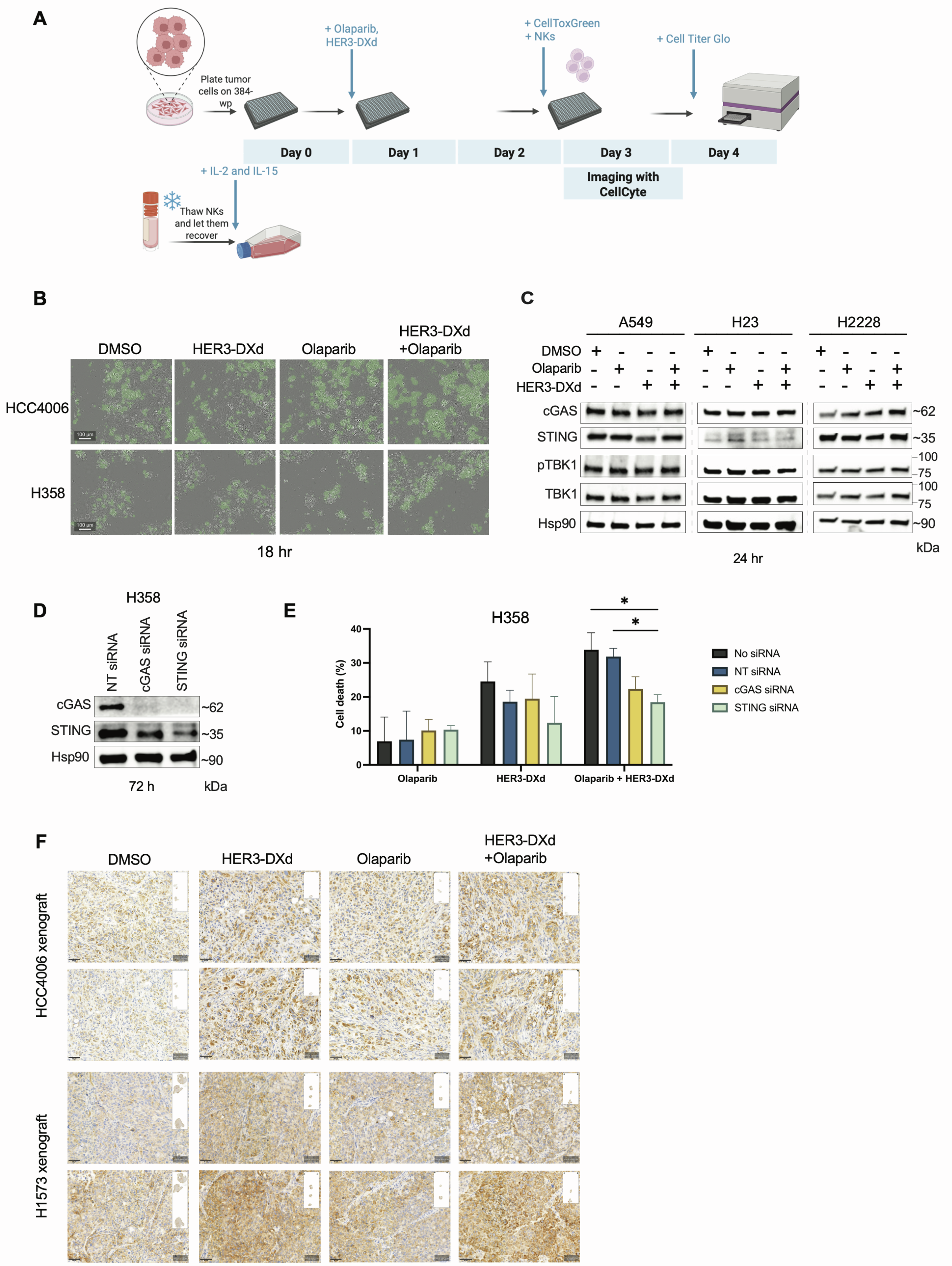

A
+ Olaparib, HER3-DXd
+ CellToxGreen
+ NKs
+ Cell Titer Glo
Plate tumor cells on 384-wp
Day 0
Day 1
Day 2
Day 3
Day 4
Imaging with CellCyte
+ IL-2 and IL-15
Thaw NKs and let them recover
B
DMSO
HER3-DXd
Olaparib
HER3-DXd +Olaparib
HCC4006
H358
18 hr
C
A549
H23
H2228
DMSO
Olaparib
HER3-DXd
cGAS
STING
pTBK1
TBK1
Hsp90
~62
~35
100
75
100
75
~90
kDa
24 hr
D
H358
NT siRNA
cGAS siRNA
STING siRNA
cGAS
STING
Hsp90
~62
~35
~90
72 h
kDa
E
H358
Cell death (%)
Olaparib
HER3-DXd
Olaparib + HER3-DXd
No siRNA
NT siRNA
cGAS siRNA
STING siRNA
F
DMSO
HER3-DXd
Olaparib
HER3-DXd +Olaparib
HCC4006 xenograft
H1573 xenograft

Figure S5: Cytotoxicity assessment and cGAS–STING pathway activation across NSCLC cell lines. (related to Figure 6). (A) Experimental design for the NK killing assay. (B) Representative images of cytotoxicity visualized with CellCyte live cell imaging and CellTox Green in *EGFR*-mutated HCC4006 and *KRAS*-mutated H358 cells after 18 hours of NK cell addition. Scale bar, 100 µm. (C) Western blot analysis of cGAS-STING pathway activation in A549, H23, and H2228 cells treated with olaparib (1 µM), HER3-DXd (10 µg/mL), or their combination, n = 3 biological replicates. Hsp90 was used as a loading control. (D) Western blot analysis of cGAS and STING expression following siRNA-mediated silencing of cGAS or STING on H358, assessed at 72 h, n = 3 biological replicates. Hsp90 was used as a loading control. (E) NK cell cytotoxicity in H358 cells after siRNA-mediated knockdown of cGAS or STING, treated with olaparib (1 µM), HER3-DXd (10 µg/mL), or their combination. Data are presented as mean ± SD from three biological replicates, with five technical replicates per biological replicate. Two-way ANOVA; *$P < 0.05$. NT, non-targeting control. (F) Immunohistochemical analysis of cGAS in paraffin-embedded HCC4006 and H1573 xenograft tissues (n = 2 per condition). Treatments included HER3-DXd (3 mg/kg), olaparib (100 mg/kg), or their combination. Scale bar, 50 µm.

Table S1: IC50 values in µM for tested inhibitors (related to Figure 1D).

	HCC4006	H1975	DFCI-243	DFCI-284	PC-9
Olaparib (PARPi)	~ 0.2289	-0.3276	~5.384e+014	0.1875	1,414
MK1775 (WEE1i)	0.2746	-1,074	0.05982	-1,310	0.103
Ceralasertib (ATRi)	1,407	-0.5089	0.4208	-0.3744	0.4342
Prexasertib (CHK1i)	~ 1.832e-017	~ -6.562	0.00093	-3,183	0.00129
Barasertib (AURKBi)	~ 20.99	-1,592	0.00256	-2,516	0.01725
CFI-402257 (MPS1i)	0.1114	-1,495	0.02856	-1,691	0.02919
Volasertib (PLKi)	~ 9.059e+028	~ -16.55	0.00337	-2,986	0.00961
Rucaparib (PARPi)	~ 0.0009312	-3,525	~ 850.5	0.6863	0.7612
MK5108 (AURKAi)	~ 0.1139	-0.9011	0.318	-0.793	0.178
Veliparib (PARPi)	0.0316	~ -2.281	~ 0.003230	~ -3.547	~ 0.2933
KU-55933 (ATMi)	1,384		~ 34177	~ -0.08551	~ 1.333
Nutlin-3a (MDM2i)	~		~ 0.000	-0.3424	~ 0.1747
NSCCHK109555 (CHK2i)	NA		~ 0.004931		
PFTb			~ 0.05238		
