# Supplementary material for: PARP inhibition enhances the antitumor activity of HER3-DXd in non-small cell lung cancer
Source: Cell Rep Med. 2026 Aug 21;7(9):103002. doi: 10.1016/j.xcrm.2026.103002 (PMC13589489; doi:10.1016/j.xcrm.2026.103002)
Supplement: Supplementary file 2 [file mmc2.pdf]

Cell Reports
Medicine

Article

PARP inhibition enhances the antitumor activity of HER3-DXd in non-small cell lung cancer

Graphical abstract

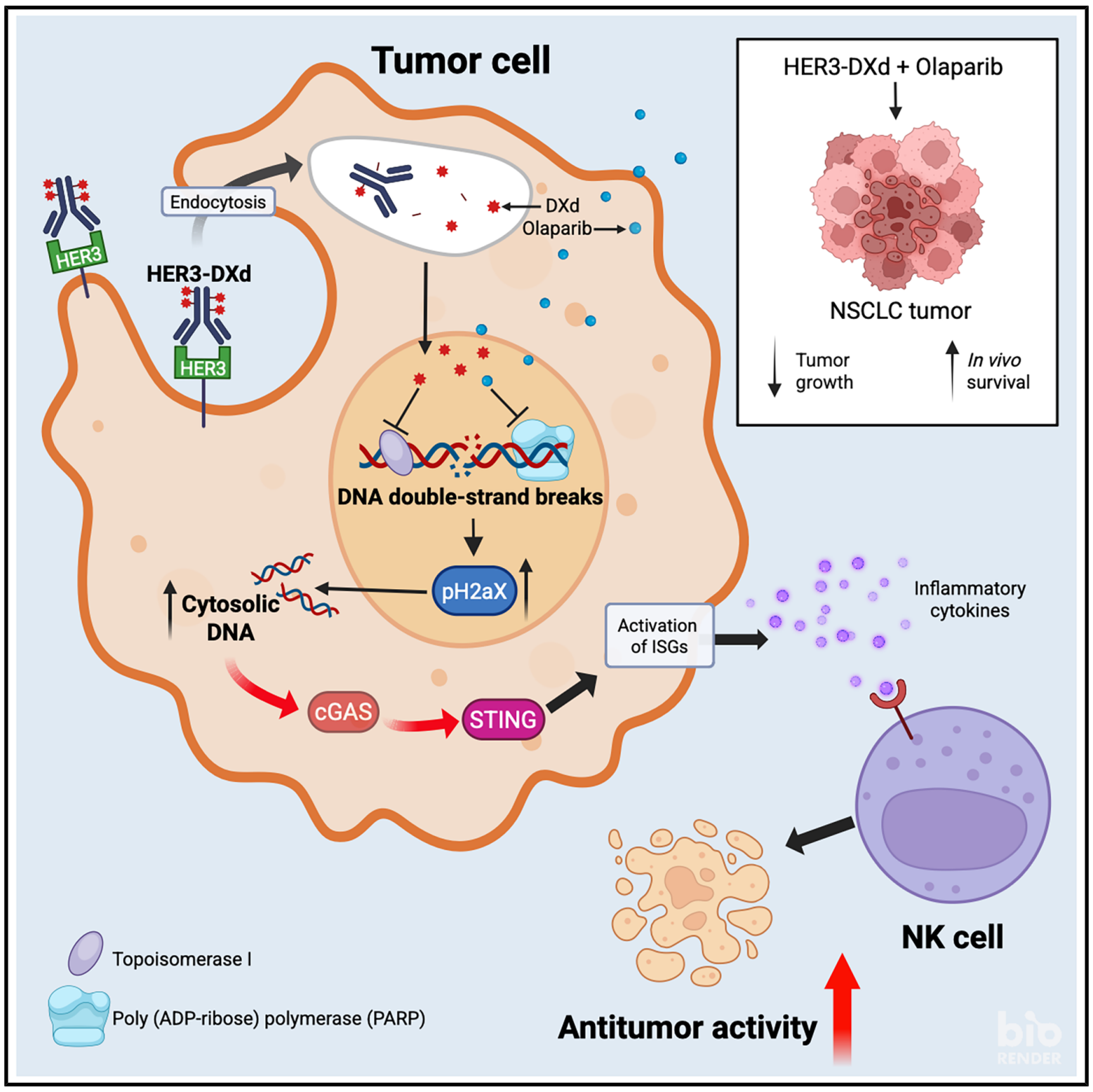

Authors

Linh Lin, Narges Moradi,
Iris A. Lähdeniemi, ..., Prafulla C. Gokhale,
Pasi A. Jänne, Heidi M. Haikala

Correspondence

heidi.haikala@helsinki.fi

In brief

Lin et al. show that PARP inhibition enhances the antitumor activity of the HER3-targeting antibody-drug conjugate HER3-DXd in non-small cell lung cancer. The combination induces DNA damage, activates cGAS-STING signaling, and promotes NK cell-mediated antitumor activity across genetically distinct NSCLC models.

Highlights

- PARP inhibition synergizes with HER3-DXd in NSCLC
- HER3-DXd and olaparib induce irreparable DNA damage and apoptosis
- The combination suppresses tumor growth and prolongs survival *in vivo*
- HER3-DXd and olaparib activate cGAS-STING and innate immunity

Lin et al., 2026, Cell Reports Medicine 7, 103002
September 15, 2026 © 2026 The Authors. Published by Elsevier Inc.
https://doi.org/10.1016/j.xcrm.2026.103002

CellPress

Cell Reports Medicine

CellPress
OPEN ACCESS

Article

PARP inhibition enhances the antitumor activity of HER3-DXd in non-small cell lung cancer

Linh Lin,[1,2,9] Narges Moradi,[1,2,9] Iris A. Lähdeniemi,[1,2,9] Hanna K. Laitinen,[1,2] Bassel Alsaed,[1,2] Lilja Lahtinen,[1,2] Timothy Lopez,[3,4] Shadi Jansouz,[1,2] Aine Knott,[3,6] Kara M. Soroko,[3,6] Ilkka Ilonen,[2,5] Prafulla C. Gokhale,[3,6] Pasi A. Jänne,[3,4,9] and Heidi M. Haikala[1,2,3,4,7,8,9,10,*]

[1]Institute of Molecular Medicine Finland (FIMM), Helsinki Institute of Life Sciences (HiLIFE) & Translational Immunology Research Program (TRIMM), Research Programs Unit, Faculty of Medicine, University of Helsinki, Helsinki, Finland
[2]iCAN Digital Precision Cancer Medicine Flagship, University of Helsinki, Helsinki, Finland
[3]Department of Medical Oncology, Dana-Farber Cancer Institute, Boston, MA, USA
[4]Harvard Medical School, Boston, MA, USA
[5]Department of General Thoracic and Esophageal Surgery, Heart and Lung Center, Helsinki University Hospital & University of Helsinki, Helsinki, Finland
[6]Experimental Therapeutics Core and Belfer Center for Applied Cancer Science, Dana-Farber Cancer Institute, Boston, MA, USA
[7]Center for Cancer Eradication Research, Faculty of Medicine and Health Technology, Tampere University, Tampere, Finland
[8]Research Unit, Heart and Lung Center, Helsinki University Hospital, Helsinki, Finland
[9]These authors contributed equally
[10]Lead contact
*Correspondence: heidi.haikala@helsinki.fi
https://doi.org/10.1016/j.xcrm.2026.103002

SUMMARY

Lung cancer, a leading cause of cancer-related mortality, is often driven by mutations in the key oncogenes epidermal growth factor receptor (*EGFR*) and Kirsten rat sarcoma virus (*KRAS*). Despite advancements of targeted therapies such as tyrosine kinase inhibitors, resistance remains a significant hurdle. Overexpression of HER3, associated with poor prognosis in non-small cell lung cancer (NSCLC), presents an alternative therapeutic target. In this study, we investigate the efficacy of the HER3-targeting antibody-drug conjugate HER3-DXd and its synergistic potential when combined with cell cycle and DNA damage response modulators. A significant synergy is observed with PARP inhibitors, effective in both *EGFR*- and *KRAS*-mutated NSCLC models. This combination markedly enhances DNA damage, induces apoptosis, and slows down *in vivo* tumor progression. Notably, this regimen also triggers antibody-dependent immunomodulatory effects through cGAS-STING pathway activation, potentiating innate immune cells for tumor killing. Our findings suggest that combining HER3-DXd with PARP inhibitors offers a promising therapeutic approach, effectively targeting diverse NSCLC subtypes.

INTRODUCTION

Non-small cell lung cancers (NSCLC) frequently harbor mutations in key oncogenes such as epidermal growth factor receptor (*EGFR*) and Kirsten rat sarcoma virus (*KRAS*).[1] Tyrosine kinase inhibitors (TKIs), such as the third-generation mutant-specific EGFR inhibitor osimertinib, have led to improvements in overall survival (OS) of NSCLC patients. Current evidence underscores the efficacy of osimertinib as the frontline therapy for *EGFR*-mutated advanced NSCLC,[2] although drug resistance remains an inevitable challenge. Recently, the mutant-specific KRAS inhibitors have received clinical approval. However, their impact on progression-free survival (PFS) remains modest as single agents, potentially attributed to emerging resistance mechanisms.[3,4] Several of these inhibitors are currently undergoing further clinical evaluation as single agents or in combinations, but their long-term curative potential is yet to be determined.

HER3 is expressed in many malignant solid tumor types, and overexpression of HER3 has been associated with poor prognosis and metastatic progression of the disease.[5,6] HER3 is not highly expressed in normal lung tissue, but 82.7% of NSCLC tumors express HER3.[7,8] The broad expression of HER3 makes it an attractive therapeutic target for NSCLC.[9] Patritumab deruxtecan (HER3-DXd, U3-1402) is an antibody-drug conjugate (ADC) containing a fully human HER3 antibody (patritumab) linked to a topoisomerase I (TOP1) inhibitor, DXd (exatecan derivative).[10] In a recently completed phase 1 study, HER3-DXd showed promising results in *EGFR*-mutated NSCLC previously treated with either chemotherapy or EGFR inhibitors (overall response rate [ORR]: 29.8%, OS: 11.9 months).[11]

We recently reported that osimertinib treatment potentiates HER3-DXd efficacy *in vitro* and *in vivo* mouse models.[12] Mechanistically, osimertinib pre-treatment increased the membrane expression of HER3, leading to elevated internalization and DNA damaging efficacy of HER3-DXd. Another study showed

© 2026 The Authors. Published by Elsevier Inc.
This is an open access article under the CC BY license (http://creativecommons.org/licenses/by/4.0/).

that HER3-DXd, in combination with the EGFR TKI erlotinib, also has potent anticancer efficacy.[13] Phase 1 clinical trial evaluating the safety and efficacy of the combination of osimertinib with HER3-DXd is currently underway (NCT04676477).

In general, ADCs feature a complex structure that integrates the specificity of monoclonal antibodies with the cytotoxicity of chemotherapeutics.[14] Their structural organization facilitates multi-level synergistic effects with other drugs to target different signaling pathways or cellular processes. In addition to their primary cytotoxic function, ADCs can exhibit a range of other activities, including antibody-dependent cellular cytotoxicity (ADCC), complement-dependent cytotoxicity (CDC), and antibody-dependent cellular phagocytosis (ADCP), as well as the bystander effect.[15,16] These diverse mechanisms of action open possibilities for innovative combination therapies.

The efficacy of the previously described combination of HER3-DXd with osimertinib is dependent on the presence of mutant *EGFR*.[12] To underpin mutation-independent synergistic effects for HER3-DXd, we screened for its rational combinations with modulators of cell cycle and DNA damage response (DDR). We identified a synergy between PARP inhibitors and HER3-DXd in different NSCLC models. Our study suggests that HER3-DXd treatment, together with PARP inhibition, serves as a potential therapeutic strategy for NSCLC and could enhance immunogenic effects of ADCs at large.

RESULTS

Combination screening identifies synergistic therapies with HER3-DXd

To discover combination therapies utilizing the distinct mechanistic properties of ADCs, such as their ability to enhance DNA damage in a targeted manner, we examined the impact of DDR and cell cycle modulators on cell viability across NSCLC cell lines (inhibitors and the experimental workflow are depicted in Figures 1A–1C). First, to evaluate the single agent potency of the drug candidates for further studies, we carried out an IC_{50} analysis using 14 inhibitors in five cell lines with various *EGFR* mutations (Figure 1D; Table S1). From these assessments, eight inhibitors were selected based on their IC_{50} values for a further synergy investigation with HER3-DXd. To analyze the effect of the combinatorial treatments, we conducted a live-cell imaging-based confluency screening over a 7-day period, comparing cell proliferation in the cell lines treated with either HER3-DXd and each inhibitor alone with the proliferation of those treated with drug combinations (Figure 1E, a higher synergy ratio indicates that the efficacy of the combination therapy is superior to that of monotherapy). Our analysis unveiled several synergies between HER3-DXd and the selected inhibitors across all tested cell lines. Notably, the combinations of HER3-DXd with olaparib (PARPi), ceralasertib (ATRi), and MK1775 (WEE1i) were most synergistic. Some synergies, such as with MK5108 (AURKAi) and CFI-402257 (MPS1i), appear to be specific to cell lines. The combination with olaparib consistently demonstrated a profound and uniform synergistic effect, with the ratio of single agent to combination therapy efficacy surpassing 1.5 across all cell lines. These findings strongly suggested that while other combinations might potentiate the effects of HER3-DXd in specific contexts, olaparib could substantially potentiate the HER3-DXd efficacy across a range of cellular backgrounds.

Lung cancer cells demonstrate sustained response to HER3-DXd and olaparib combination treatment despite their genetic background

To explore the long-term efficacy of HER3-DXd combined with olaparib, we evaluated their impact on three *EGFR*-mutant cell lines (HCC4006, H1975, and DFCI-284), using live-cell imaging. Over an extended observation period, our findings consistently showed a strong combination response across all three cell lines (Figures 2A–2C). Apoptosis, measured using a cleaved-caspase-3/7 assay, revealed enhanced apoptotic induction with the combination treatment compared with single-agent olaparib or HER3-DXd (Figure 2D). Protein analysis confirmed HER3 expression in all three cell lines (Figures 2E and 2F). To assess differences in potential sensitivity to various PARP inhibitors, we tested the synergy of HER3-DXd with seven different PARP inhibitors, measuring both cell confluency and apoptosis (Figures S1A–S1D). All were synergistic with HER3-DXd, with talazoparib and niraparib leading to the strongest synergy. However, because olaparib is the furthest along in clinical utilization and also resulted in good synergy, we decided to proceed with olaparib for the remainder of our study.

As HER3 is widely expressed in tumors with various genetic backgrounds, the effect of HER3-DXd combined with olaparib should extend beyond *EGFR*-mutant cells. Therefore, we aimed to further investigate the potency of the HER3-DXd and olaparib combination in a *KRAS*-mutant setting, another dominant oncogenic driver in NSCLC. Because the tested lung cancer cell lines did not reach IC_{50} values for olaparib (Table S1, related to Figure 1D), higher olaparib concentrations with a fixed HER3-DXd dose were used to ensure sufficient target inhibition. While analyzing HER3 expression levels in *KRAS*-mutant cell lines, we found that most cell lines expressed HER3 (Figures 2E and 2F). Next, the cells with different *KRAS* mutations were treated with HER3-DXd, olaparib, and the combination of both agents. We observed clear synergistic effects with the combination in two cell lines (H1373 and H1573), while other cell lines (H358 and LCLC-97TM1) were already highly responsive to HER3-DXd alone (Figures 2G–2J). In H358 cells, where HER3-DXd was highly effective as a single agent, HER3 expression was notably higher than those in the other cell lines.

The relationship between target expression and ADC activity does not always follow a linear pattern and is highly dependent on the ADC and its target. In our experiments, the HER3-negative/low cell lines A549 and H23 did not respond to either the single-agent HER3-DXd or its combination with olaparib (Figures 2K and 2L), indicating that HER3 expression is important for the baseline efficacy of HER3-DXd and its combinations. However, H2228, which is negative for both *KRAS* and *EGFR* mutations and had low level of HER3, did not respond to single-agent HER3-DXd, but it responded to the HER3-DXd and olaparib combination that induced a synergetic effect (Figure S1F), suggesting that the observed combination benefit is independent of the mutational status.

Overall, our data support the conclusion that HER3 expression can influence the efficacy of the HER3-DXd and olaparib

A

Drug name	MK5108	Barasertib	Volasertib	CFI-402257	Olaparib	Rucaparib	Veliparib	KU-55933	NSC 109555 ditosylate (CHK2i)	Ceralasertib	Prexasertib	MK1775	PFT-β	Nutlin-3a
**Drug target**	AURKA	AURKB	PLK1	MPS1	PARP	PARP	PARP	ATM	CHK2	ATR	CHK1	WEE1	p53	MDM2

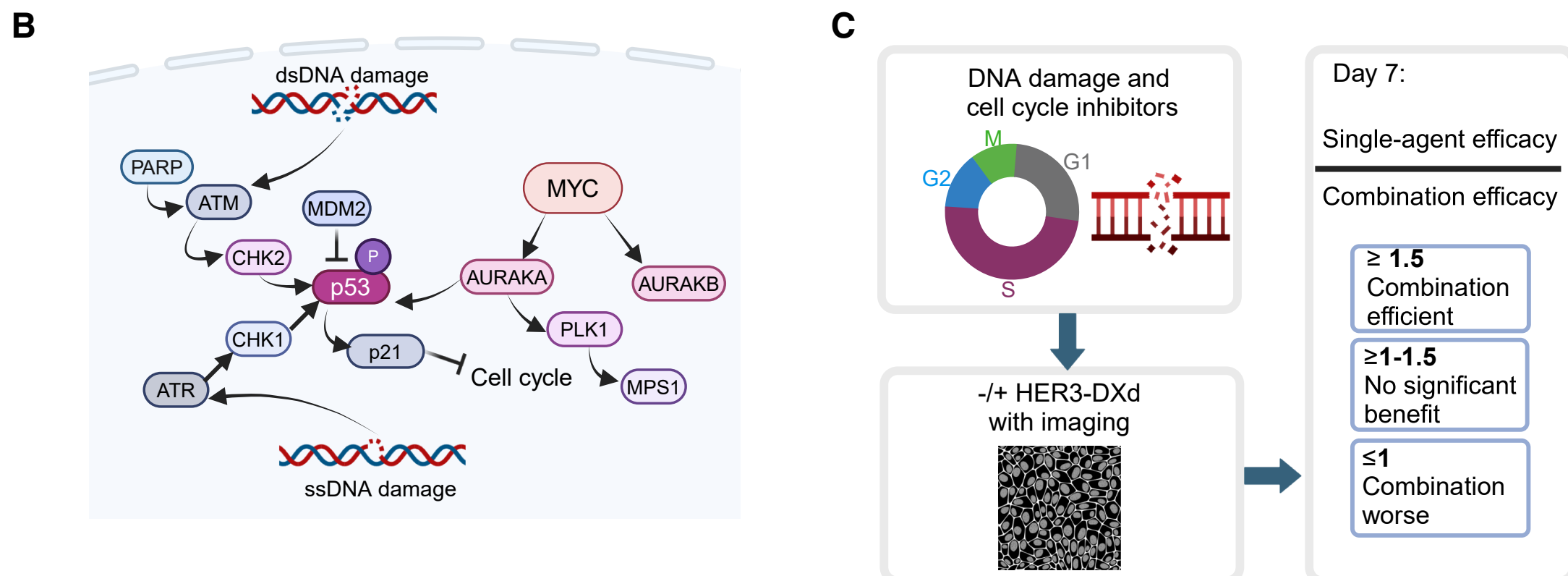

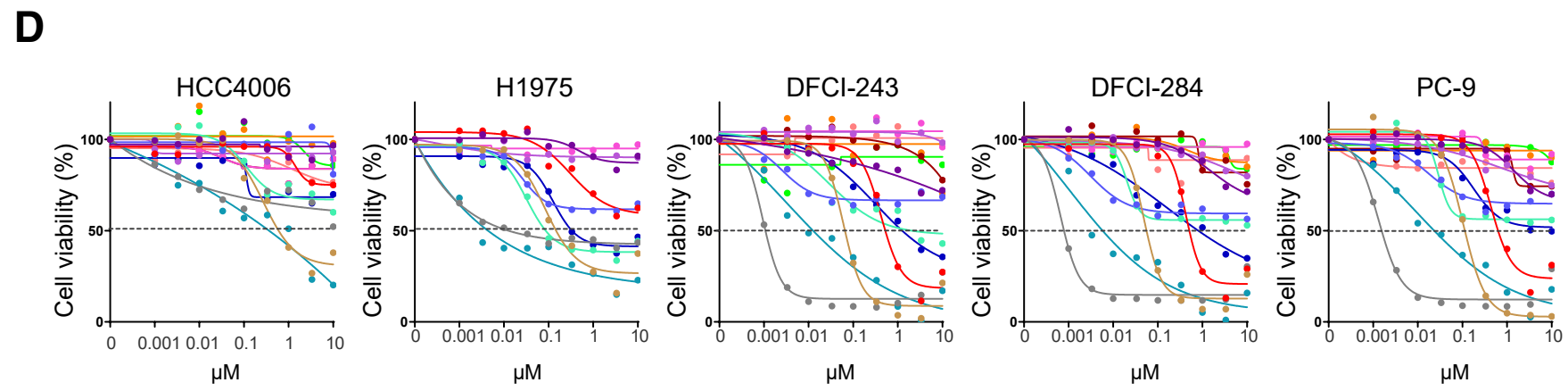

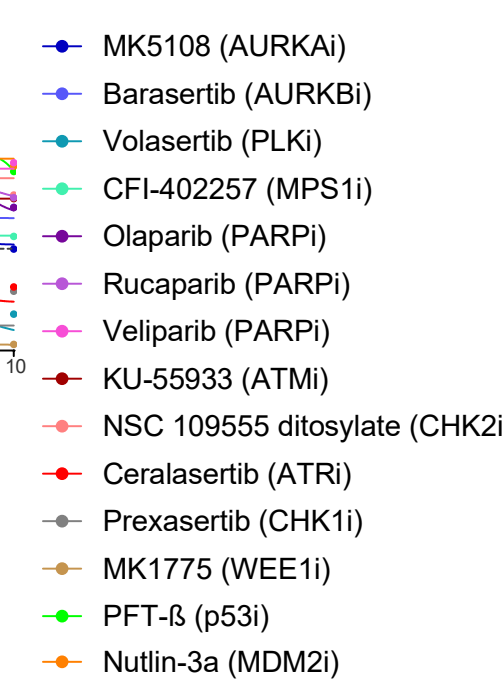

E

	MK5108	Barasertib	Volasertib	CFI-402257	Olaparib	Ceralasertib	Prexasertib	MK1775
HCC4006	0.80	1.17	1.07	2.27	2.32	1.56	1.00	1.66
H1975	0.95	1.06	0.74	0.97	1.91	1.98	2.64	2.41
DFCI-243	1.73	0.95	1.30	0.83	1.66	1.11	1.00	0.93
DFCI-284	1.05	0.98	1.46	0.95	2.03	2.19	1.95	1.46
PC-9	1.59	0.91	1.34	0.94	3.29	1.90	1.17	1.47
	AURKAi	AURKBi	PLK1i	MPS1i	PARPi	ATRi	CHK1i	WEE1i

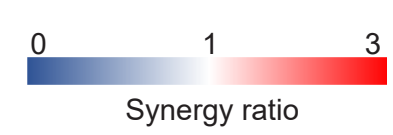

Figure 1. Overview of the combination screening approach to identify synergistic therapies with HER3-DXd in NSCLC cell lines
(A) List of the 14 initially included DDR and cell cycle modulators, along with their respective molecular targets.
(B) Overview of the molecular mechanisms of the DDR and cell cycle modulators targeted in the study.
(C) Detailed experimental workflow assessing the impact of the included inhibitors on cell proliferation.
(D) Dose-response analysis of the 14 inhibitors across five NSCLC cell lines with different *EGFR* mutations. IC_{50} values were determined from three independent experiments. Drug concentration for the next step of the screening was selected based on the IC_{50} value. See also Table S1.
(E) Synergy heatmap from the 7-day live-cell imaging-based confluency screening, showing the comparison of cell confluency between treatments with inhibitors alone and those with the inhibitor and HER3-DXd combination.

combination therapy, but it may not be the sole determining factor in both *EGFR*- and *KRAS*-mutant cells.

HER3-DXd synergizes with olaparib in tumor-on-chip assay

To investigate the synergy between HER3-DXd and olaparib, tumor-on-chip assays were conducted using patient-derived lung cancer organoids (ORG-29: *EGFR* L858R; LUNG-31: *EGFR* E19del; and ORG-54: *KRAS* G12S, validated with exome sequencing) embedded in the middle channel of a microfluidic chip, with endothelial tubules built on the proximal side channel to mimic the tumor vasculature. This design allowed for precise delivery of the single agents or their combination through the endothelial tubule, simulating *in vivo* drug distribution through the vascular system (Figure 3A).

To assess cell viability and the extent of cell death, live/dead-cell analysis was performed using acridine orange (AO) and propidium iodide (PI) staining (Figures 3B–3D). The results showed that

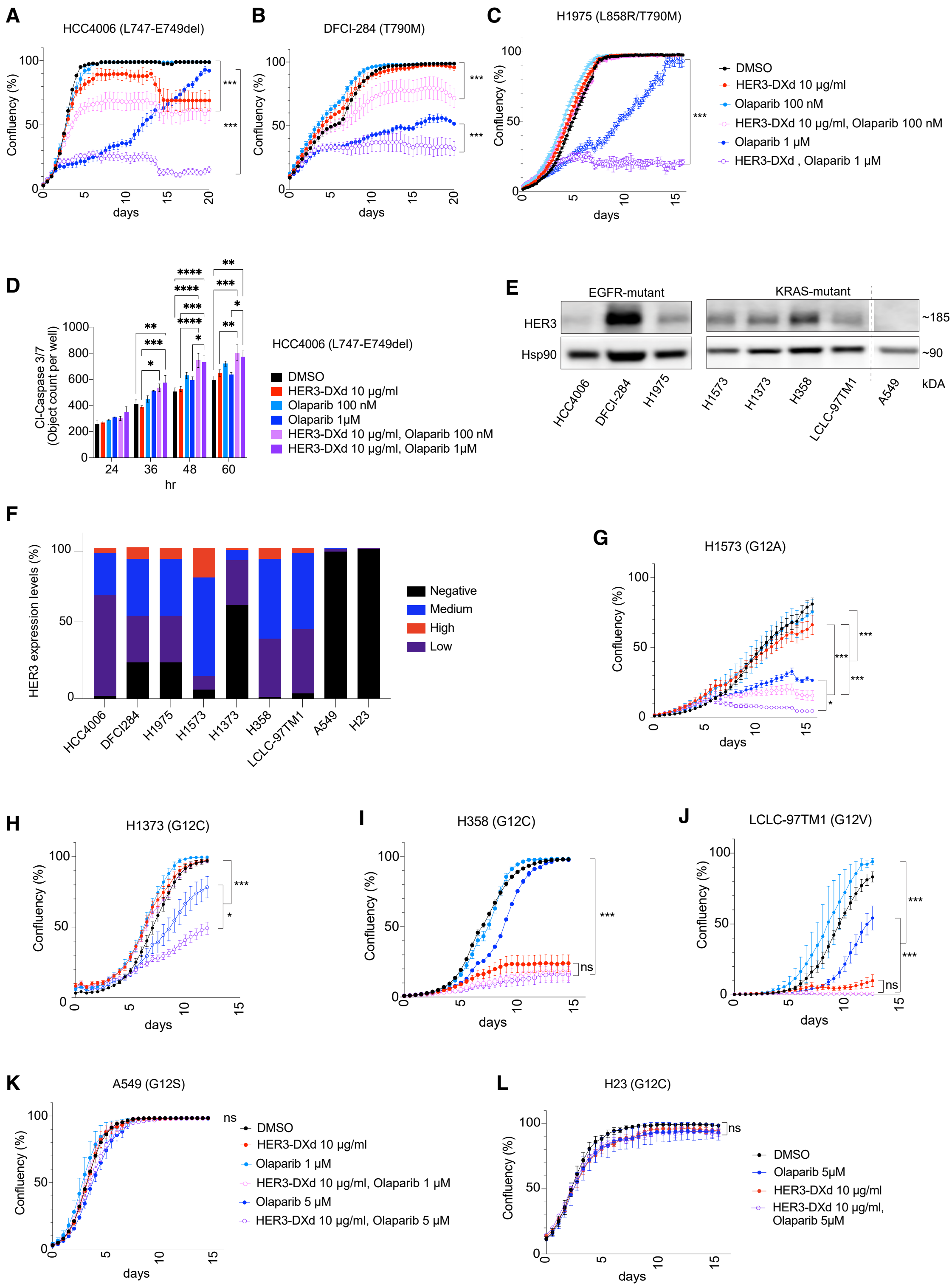

Figure 2. Long-term response to HER3-DXd and olaparib in *EGFR*- and *KRAS*-mutant cell lines

(A–C) Live imaging-based proliferation assays for efficacy of the HER3-DXd and olaparib combination in the *EGFR*-mutant cell lines HCC4006, H1975, and DFCI-284. 10 μg/mL HER3-DXd and 100 nM/1 μM olaparib were used. Data are presented as mean ± SEM and are representative of three independent experiments. Two-way ANOVA.

(legend continued on next page)

while single-agent HER3-DXd and olaparib induced cell death only in the *KRAS*-mutant model, HER3-DXd + olaparib significantly impacted cell viability in both *EGFR*- and *KRAS*-mutant settings (Figures 3E–3G). These results suggest that drug efficacy may be greater in cell lines with high HER3 membrane expression than in those expressing medium or low levels (Figure 3H). This finding provides compelling *ex vivo* evidence for the combination therapy's ability to target tumors more effectively.

HER3-DXd and olaparib combination treatment leads to DNA damage beyond repair

HER3-DXd is composed of an HER3 antibody conjugated to a derivative of a TOP1 inhibitor, which inhibits TOP1 function, leading to DNA single-strand breaks (SSBs) and potential cell death if not repaired. Moreover, olaparib inhibits PARP, preventing the repair of these SSBs. Olaparib also traps PARP on the DNA, further exacerbating DNA damage by preventing the release of PARP from the DNA. This results in the accumulation of DNA damage and drives cells toward DDR-induced cell death, particularly in cells already vulnerable to impaired DNA repair mechanisms (Figure 4A). To investigate how the synergistic treatment of cells with HER3-DXd and olaparib modulates the DDR, we further analyzed DDR in the drug-treated cells.

In an *EGFR*-mutant setting (HCC4006), pATM and pH2aX levels increased after 48 h of HER3-DXd or HER3-DXd + olaparib treatment (Figure 4B). Additionally, the p21 cell cycle inhibitor was upregulated by the combination treatment. pATM and pH2aX were also upregulated by the combination treatment in *KRAS*-mutant setting (H358) (Figure 4C). Flow cytometry analysis of pH2aX and pATM confirmed the previous findings; the number of highly expressing cells was significantly increased in HER3-DXd + olaparib-treated cells compared with single-agent-treated cells (Figure 4D; Figures S2A–S2C).

DNA damage is a well-established regulator of the cell cycle. To assess the impact of these treatments on cell cycle progression, the cells were stained with PI and analyzed by flow cytometry. In both *EGFR*- and *KRAS*-mutant settings, the combination of HER3-DXd and olaparib induced G2/M phase cell-cycle arrest (Figures 4E and 4F). Collectively, these results suggest that the combination treatment causes irreparable DNA damage, halts cell-cycle progression, and ultimately drives cells toward apoptosis.

In vivo treatment with HER3-DXd and olaparib combination slows down tumor growth and prolongs survival

To evaluate the therapeutic impact of the HER3-DXd and olaparib combination on tumor growth *in vivo*, we performed a series of experiments using NSG mice injected subcutaneously with *EGFR*-mutant HCC4006 cells and *KRAS*-mutant H1573 cells. The mice were randomly assigned to four treatment groups: vehicle control group, olaparib alone group, HER3-DXd alone group, and a HER3-DXd and olaparib combination group. Tumor growth was monitored twice a week, with mice being sacrificed when tumor volumes reached 2,000 mm^3, in accordance with ethical guidelines for animal welfare (Figure 5A). This approach allowed us to closely monitor both the efficacy of the individual treatments and the combination therapy over time. The concentration of the HER3-DXd was chosen based on an earlier study.[12]

Interestingly, while single-agent HER3-DXd was effective in HCC4006, the combination of HER3-DXd with olaparib led to a marked reduction in tumor growth in both *EGFR*- and *KRAS*-mutant models (Figures 5B–5E). The combination treatment not only slowed tumor progression significantly but also appeared to exert a prolonged effect on the tumor growth rate. Notably, at the end of the treatment period, tumors in the combination-treated group were significantly smaller than those in the single-agent groups (Figures 5B–5E). Furthermore, survival analysis demonstrated a significant extension in the lifespan of both the *EGFR*-mutant and *KRAS*-mutant mouse models receiving the combination therapy (Figures 5F and 5G).

Systemic toxicity was assessed by monitoring body weight (Figures S3A–S3C) and evaluating the hematological and serum chemistry parameters (Figures S4A–S4D). Treatment with HER3-DXd alone at doses of 3 and 10 mg/kg, as well as the combination of HER3-DXd with olaparib (with HER3-DXd administered at 3 mg/kg to evaluate potential synergistic effects), did not result in significant body weight loss or other overt signs of toxicity (Figures S3A–S3C). Complete blood count (CBC) analysis revealed a modest decrease in lymphocyte percentage in the olaparib-treated group, whereas HER3-DXd monotherapy and the combination treatment did not induce notable hematological changes (Figures S4A and S4B). No statistically significant differences in CBC parameters were observed between the control and treatment groups. Similarly, serum chemistry analyses performed on day 10 did not reveal significant treatment-related alterations (Figures S4C and S4D). However, at a later assessment (day 24), increased alanine transaminase (ALT) levels were observed in the combination treatment group, suggesting a potential hepatic effect associated with prolonged treatment. Despite this finding, no accompanying body-weight loss or overt clinical signs of toxicity were detected. Collectively, these results indicated that HER3-DXd monotherapy and the combination regimen were generally tolerated under the dosing

(D) Apoptosis assays, marked by cleaved-caspase-3/7 activity, comparing apoptotic induction in the control, single-agent treatments, and combination treatments. 10 μg/mL HER3-DXd and 100 nM/1 μM olaparib were used. Data are presented as mean ± SD and are representative of three independent experiments. One-way ANOVA.

(E) Western blot analysis of HER3 in *EGFR*- and *KRAS*-mutant cell lines; $n = 3$ biological experiments.

(F) Flow cytometry quantification of HER3 expression levels in HCC4006, DFCI-284, H1975, H1573, H1373, H358, LCLC-97TM1, A549, and H23. $n = 3$ biological experiments.

(G–L) Live imaging-based proliferation assays for efficacy of the HER3-DXd and olaparib combination in the *KRAS*-mutant cell lines H1373, H1573, H358, LCLC-97TM1, A549, and H23. 10 μg/mL HER3-DXd and 1 μM/5 μM olaparib were used. Data are presented as mean ± SD and are representative of three independent experiments. One-way ANOVA. *$p < 0.05$; **$p < 0.01$; ***$p < 0.001$; and ****$p < 0.0001$; ns, not significant.
See also Figure S1.

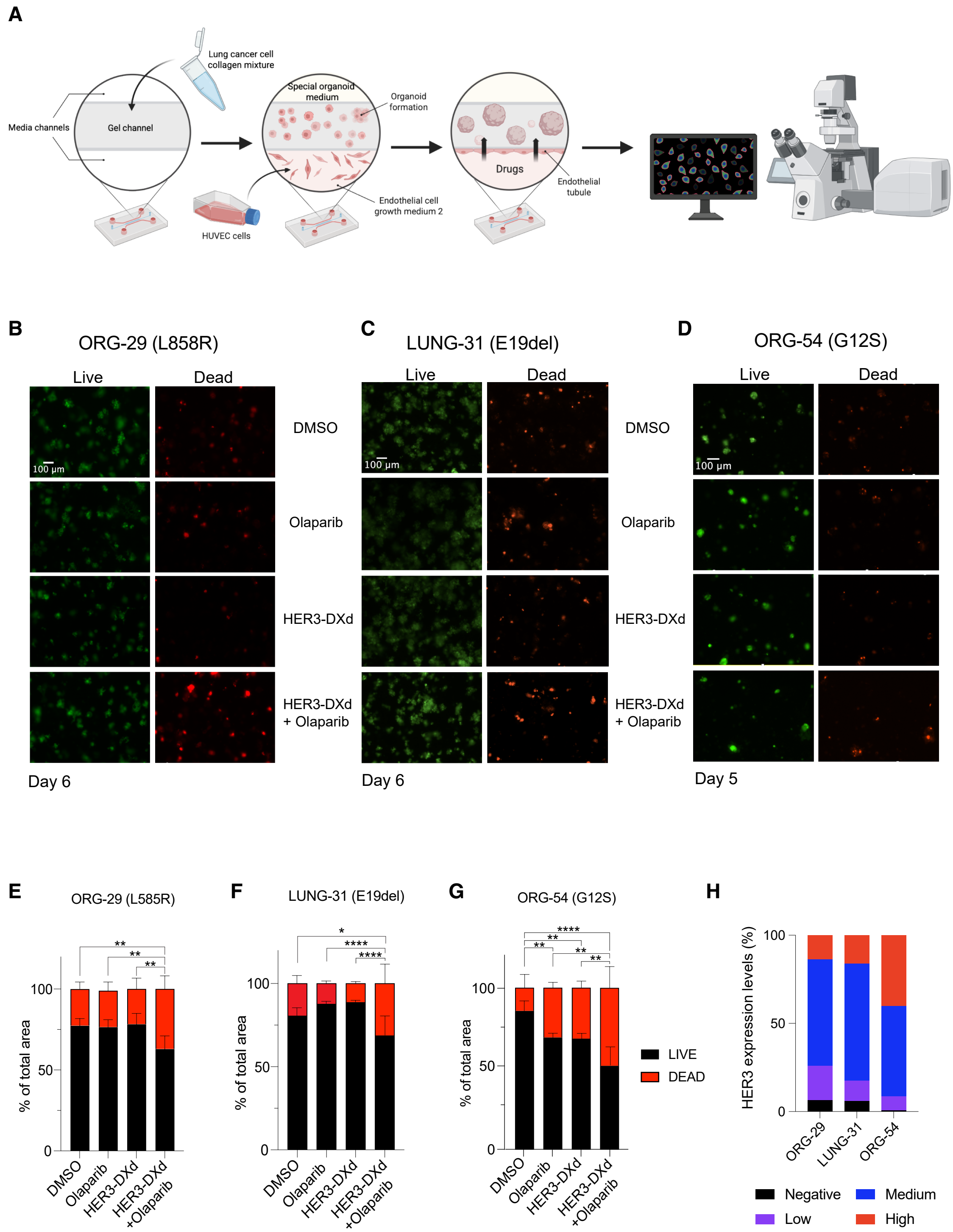

Figure 3. HER3-DXd and olaparib response in patient-derived tumor-on-chip models

(A) A schematic overview of the tumor-on-chip assay. Patient tumor-derived organoid cells were seeded into the middle channel forming tumor organoids, whereas an endothelial tubule was formed in the proximal side channel. After the formation of the endothelial tubule, the drugs were administered to the chip through the artificial vasculature. The models were AO/PI stained after 5–6 days of treatment, then imaged.

(B–D) Representative live/dead images of tumor-on-chip *EGFR* and *KRAS* models with AO/PI staining at the end of the drug treatments. ORG-29 and LUNG-31 were analyzed on day 6, and ORG-54 was analyzed on day 5. 20 µg/mL HER3-DXd and 2 µM olaparib were used. Scale bars, 100 µm.

(legend continued on next page)

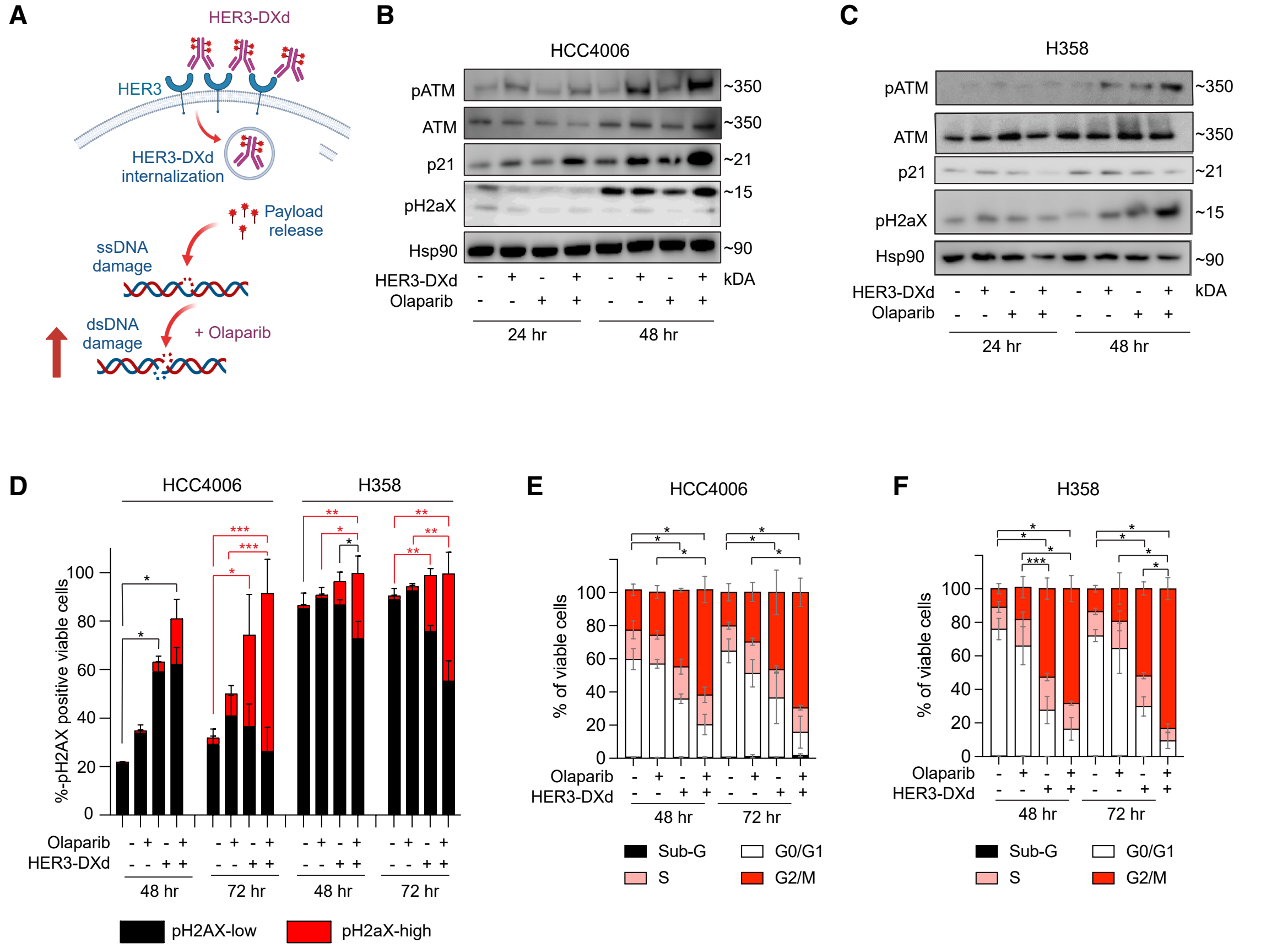

Figure 4. Activation of DNA damage response in *EGFR*- and *KRAS*-mutant cells following the treatment with HER3-DXd and olaparib

(A) Hypothesized mechanism of DNA damage in HER3-DXd and olaparib combination therapy.

(B and C) Representative western blot of DNA damage marker levels in the *EGFR*-mutant cell line HCC4006 and the *KRAS*-mutant cell line H358 treated with or without HER3-DXd and olaparib combinations; $n = 3$ biological experiments. 10 μg/mL HER3-DXd and 1 μM olaparib were used.

(D) Flow cytometry-based analysis of pH2aX levels in the *EGFR*-mutant cell line HCC4006 and the *KRAS*-mutant cell line H358 treated with or without HER3-DXd and olaparib combinations; $n = 3$ biological experiments. 10 μg/mL HER3-DXd and 1 μM olaparib were used. Data are presented as mean ± SD. One-way ANOVA.

(E and F) Flow cytometry-based analysis of cell cycle in the *EGFR*-mutant cell line HCC4006 and the *KRAS*-mutant cell line H358 treated with HER3-DXd and olaparib alone or in combination for 48 or 72 h. 10 μg/mL HER3-DXd and 1 μM olaparib were used; $n = 3$ biological experiments. Data are presented as mean ± SD. One-way ANOVA. *$p < 0.05$; **$p < 0.01$; ***$p < 0.001$; and ****$p < 0.0001$; ns, not significant.

See also Figure S2.

conditions tested, although the observed ALT elevation warrants further investigation and monitoring in future studies.

Our results underscore the potential of the HER3-DXd and olaparib combination to not only reduce tumor growth but also enhance long-term survival, a crucial endpoint in cancer therapy.

HER3-DXd along with olaparib induces antitumor immune activation via cGAS-STING

A critical mechanism for activating innate immunity in cancer is the detection of cytosolic DNA by cyclic GMP-AMP synthase (cGAS), which activates the stimulator of interferon genes (STING) pathway, ultimately triggering an immune response.[17] When STING is activated, it initiates a signaling cascade that leads to the production of type I interferons and other pro-inflammatory cytokines, enhancing immune cell recruitment and promoting an antitumor immune response. We hypothesized that the combination of HER3-DXd with olaparib could enhance immune cell killing across different cancer cell lines, particularly through dsDNA damage and cytosolic DNA release (Figure 6A).

To investigate this, we first examined the natural killer (NK) cell-mediated killing effects of HER3-DXd and olaparib on two cell lines: *EGFR*-mutated HCC4006 and *KRAS*-mutated H358.

(E–G) Live/dead analysis from tumor-on-chip *EGFR* (ORG-29 and LUNG-31) and *KRAS* (ORG-54) models. $n = 5$ biological replicates in (E) and (F) and $n = 6$ biological replicates in (G). 20 μg/mL HER3-DXd and 2 μM olaparib were used. Data are presented as mean ± SD and were analyzed using two-way ANOVA, with Tukey's multiple-comparisons test. *$p < 0.05$; **$p < 0.01$; ***$p < 0.001$; and ****$p < 0.0001$; ns, not significant.

(H) Flow cytometry quantification of HER3 expression levels in ORG-29, LUNG-31, and ORG-54 cell lines.

A

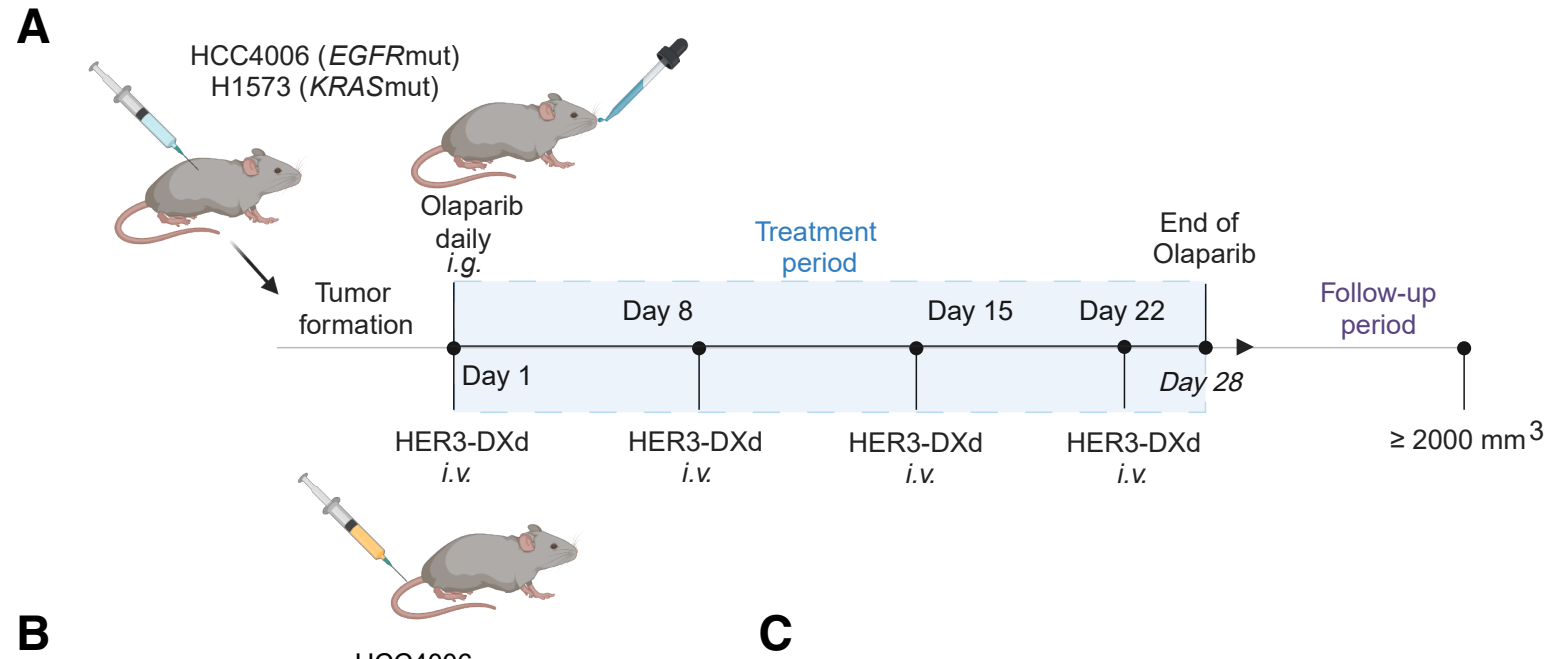

B

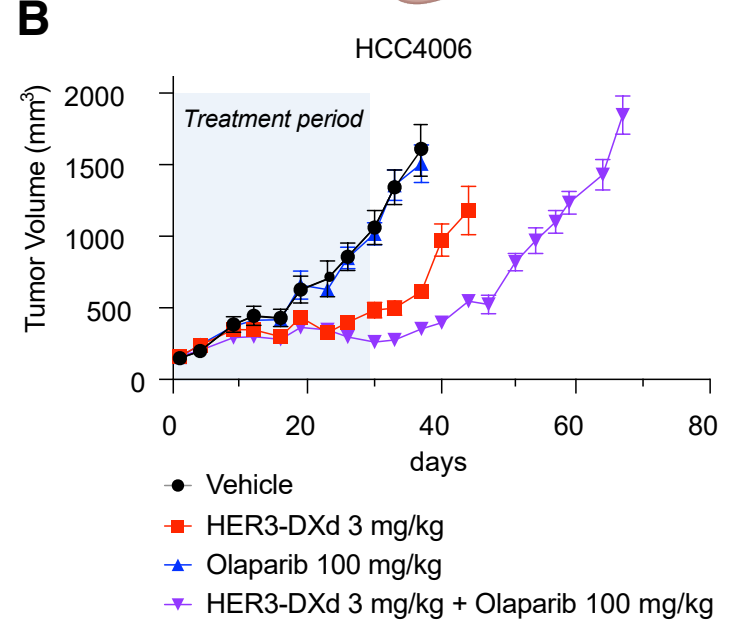

C

HCC4006 day 33	Average tumor volume (mm³) after treatment	P-value (vs. Vehicle)	P-value (vs. Olaparib)	P-value (vs. HER3-DXd)
Vehicle	1342.8	-	-	-
HER3-DXd 3 mg/kg	498.0	0.0002, ***	0.0001, ***	-
Olaparib 100 mg/kg	1357.6	>0.9999, ns	-	-
HER3-DXd 3 mg/kg +Olaparib 100 mg/kg	276.6	<0.0001, ****	0.0001, ***	0.0166, *

D

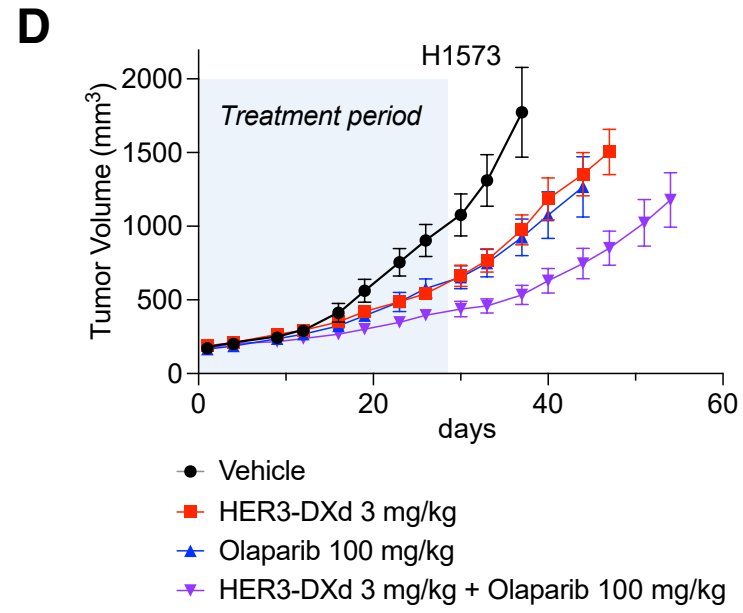

E

H1573 day 33	Average tumor volume (mm³) after treatment	P-value (vs. Vehicle)	P-value (vs. Olaparib)	P-value (vs. HER3-DXd)
Vehicle	1310.3	-	-	-
HER3-DXd 3 mg/kg	768.4	0.0923, ns	>0.9999, ns	-
Olaparib 100 mg/kg	750.2	0.0867, ns	-	-
HER3-DXd 3 mg/kg +Olaparib 100 mg/kg	459.2	0.0084, **	0.1043, ns	0.0329, *

F G

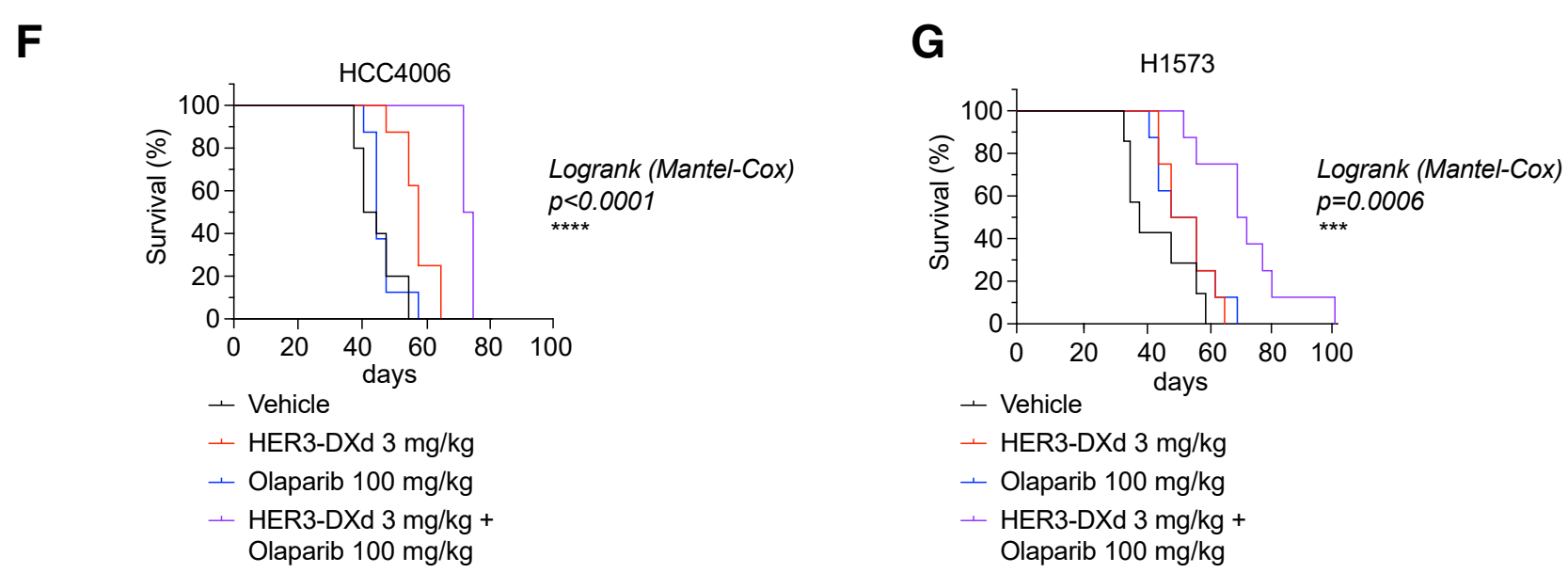

Figure 5. Impact of HER3-DXd and olaparib combination on tumor growth and survival

(A) Schematic of the workflow and timeline for tumor induction and *in vivo* treatment, detailing the administration of vehicle, olaparib, HER3-DXd, and the combination of HER3-DXd with olaparib; 8 mice were included in each treatment group.

(B–E) Analysis of tumor volume across vehicle control, single-agent treatment, and combination therapy groups on day 33. Data are presented as mean ± SEM. Brown-Forsythe and Welch one-way ANOVA. The tumor growth differences were evaluated at the end of the treatment period.

(F and G) HER3-DXd with olaparib improves overall survival in mice. $^{*}p < 0.05$; $^{**}p < 0.01$; $^{***}p < 0.001$; and $^{****}p < 0.0001$; ns, not significant. Log-rank (Mantel-Cox) test.

See also Figures S3 and S4.

Both cell lines were pre-treated for 2 days with HER3-DXd and olaparib, either alone or in combination, followed by the addition of NK cells, using two different effector-to-target ratios. Cytotoxicity assays were performed to assess NK cell-driven tumor cell death where the cell viability was quantified based on fluorescence (Figures 6B–6E), imaged with CellCyte (Figures S5A and S5B). In HCC4006 and H1975 cells, there was a moderate but significant increase in NK cell-mediated killing, particularly with the combination of HER3-DXd and olaparib (Figures 6B and 6C). In contrast, H358 cells exhibited a much stronger, NK cell dose-dependent cytotoxic response under the same treatment conditions, suggesting that these cells were more susceptible to NK cell-mediated killing, especially with the combination treatment (Figure 6D). In H1573, this immunogenic effect was only mild and barely detectable (Figure 6E).

Given the observed NK cell effects, we next explored whether HER3-DXd and olaparib activated the innate immune response via cGAS-STING. To assess this, we examined the activation of key markers involved. Intriguingly, upon treatment with HER3-DXd or with the combination of HER3-DXd and olaparib, we observed striking upregulation of cGAS expression in the HCC4006 and H358 cells (Figure 6F). In H1975 cells, we observed an upregulation of STING, suggesting a feedback upregulation of STING and that the pathway effects might be time point dependent. Even in H1573 cells with the lowest NK cell killing effect, the cGAS activation was mild yet synergistic with the HER3-DXd and olaparib combination. Similarly, in H358 cells, we observed activation of the downstream signaling of STING, demonstrated by the increased pTBK1 levels, whereas in the other cell lines, it was not as clear. In the HER3-negative/low lines A549 and H23, the combination was not able to increase cGAS, STING, or pTBK1 (Figure S5C). In H2228 cell line, which is negative for *KRAS* or *EGFR* mutation but has low HER3 expression, the cGAS effect seemed synergistic, similar to that observed with the combination efficacy earlier.

To assess whether the cGAS-STING pathway contributes causally to the observed synergy, we compared NK cell-mediated cytotoxicity following small interfering RNA (siRNA)-mediated silencing of cGAS and STING. Silencing of either component reduced NK cell killing, with STING silencing resulting in a significant decrease in cytotoxicity (Figures S5D and S5E). These findings indicated that the pathway contributes to the enhanced

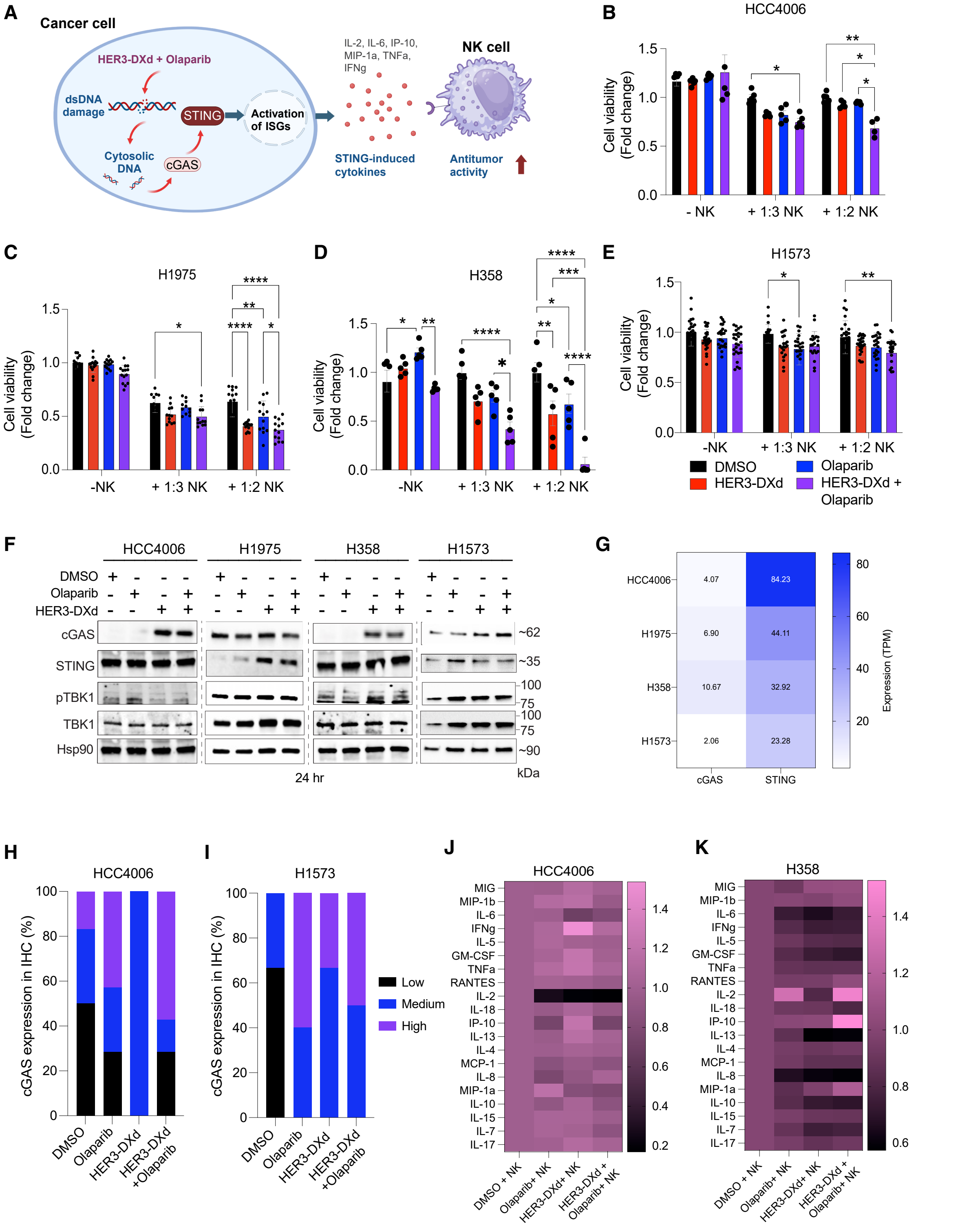

Figure 6. The effect of HER3-DXd and olaparib combination therapy on NK cell-mediated antitumor immunity

(A) Schematic of the hypothesized mechanism underlying combination treatment-driven STING activation, leading to increased antitumor activity.

(B–E) CellTiter Glo analysis of NK cell-mediated cytotoxicity, showing changes in cell viability in *EGFR*-mutant (HCC4006 and H1975) and *KRAS*-mutant (H358 and H1573) cancer cells treated with olaparib, HER3-DXd, or the combination and co-cultured with NK cells at 1:2 and 1:3 ratios. 10 μg/mL HER3-DXd and 1 μM olaparib were used 48 h prior to NK cell addition, and the NK cell killing was measured for 24 h.

(B and D) Data are representative of three independent biological experiments, with five technical replicates per condition.

(legend continued on next page)

killing effect, although it is not solely responsible, consistent with the presence of multiple cytotoxic mechanisms in NK cells.

Next, we compared baseline cGAS-STING activity in the cell lines by using publicly available Cancer Cell Line Encyclopedia (CCLE) data[18,19] and observed that H1573 had the lowest baseline cGAS-STING levels, suggesting that there might be a threshold that needs to be surpassed for the effects of the combination to properly manifest (Figure 6G).

cGAS activation induced by HER3-DXd was also observed *in vivo* in mouse models; in HCC4006 tumors, the effect appeared synergistic, whereas in H1573 tumors, HER3-DXd alone was sufficient to induce cGAS activation, with no further increase observed at the analyzed time point (Figures 6H and 6I; Figure S5F).

Activation of STING eventually leads to the activation of interferon-stimulated genes (ISGs) and, subsequently, the secretion of cytokines, including type I interferon-activated cytokines such as IP-10 (CXCL10), MCP-1 (CCL2), and MIG (CXCL9), as well as other pro-inflammatory cytokines like TNF-α and IL-6.[20] These cytokines contribute to the downstream immune response, further enhancing the antiviral and antitumor activities associated with STING activation. To assess secretome alterations following the combination treatment with NK cell stimuli, we performed multiplex cytokine profiling on the cell culture medium (Figures 6J and 6K). Specifically, in H358 cells, the combination of HER3-DXd and olaparib significantly upregulated the secretion of key STING-related immune cytokines, including IL-2, IP-10, and MIP-1α, compared with the single-agent treatments. However, this effect was absent in HCC4006 cells, suggesting impaired STING signaling. This discrepancy further highlights the variability in STING pathway activation across different cell lines, with potential implications for therapeutic strategies targeting the pathway.

Our findings indicate that HER3-DXd, when combined with olaparib, can activate the cGAS-STING pathway, promoting a potent antitumor immune response. This may enhance the antitumor immune activity, potentially improving therapeutic outcomes in HER3-expressing tumors.[19]

DISCUSSION

HER3, a member of the EGFR family, is widely expressed in NSCLC tumors, and its overexpression has been linked to resistance to various cancer therapies, particularly those targeting *EGFR* mutations. Therefore, HER3-targeted therapies are a promising strategy to overcome these resistance mechanisms in NSCLC.[9] Targeting is possible with HER3-DXd, an ADC containing TOP1 inhibitor, and currently under investigation in several clinical trials (e.g., NCT06172478 and NCT05338970). We recently reported that the treatment of lung cancer cells with HER3-DXd together with osimertinib is an efficient combinatorial treatment and leads to DNA damage in cells, and the efficacy of this combination is currently being investigated in a further clinical trial.[9] Single-agent clinical benefits with HER3-DXd have already been reported, particularly in heavily pretreated patients with *EGFR*-mutant tumors.[21,22]

To expand our knowledge about additional combinational prospects for HER3-DXd, we aimed to investigate if the DDR or cell-cycle modulation could have a synergistic effect with HER3-DXd. We showed that the treatment with a PARP inhibitor together with HER3-DXd leads to a HER3-dependent inhibition of cell proliferation, DNA damage, and prolonged survival *in vivo*.

The HER3-dependent effects of HER3-DXd plus olaparib may be influenced by HER3 expression levels, intrinsic resistance to the DXd payload, and variability in sensitivity to PARP inhibition. In breast cancer, determinants of differential response to HER3-DXd have been more extensively investigated, and RNA- and DNA-based signature analyses have been proposed to support patient selection.[23]

Notably, previous studies have demonstrated the effectiveness of TOP1 inhibitor and PARP inhibitor combinations in preclinical cancer models, both in homologous recombination-deficient (HRD) and -proficient settings.[24] Our study extends this concept to HER3-targeted ADCs in genetically defined lung cancer models, revealing disease- and target-specific determinants of response that have not been addressed in prior studies. These studies provide a strong rationale for the observed synergy between HER3-DXd and olaparib in our models, suggesting that the combination exploits DNA damage accumulation regardless of HRD status.

We, thus, propose a clinically relevant combination for HER3-DXd therapy in combination with olaparib for NSCLC treatment that could be stratified based on the presence of HER3 expression. While this combination has not yet been clinically explored in NSCLC, a similar idea is currently being investigated in breast cancer patients who have progressed on HER2-targeted T-DXd therapy (NCT06298084). The results of this trial may provide insights into the broader applicability of HER3-DXd and PARP inhibitor combinations across tumor types.

While the role of HER3 in antitumor immunity remains unclear, combining HER3-DXd with immune checkpoint inhibitors, such as anti-PD-1, has been shown to enhance tumor growth

(C) Data are pooled from three independent biological experiments, with four technical replicates per experiment (n = 12 technical replicates).
(E) Data are pooled from five independent biological experiments, with five technical replicates per experiment (n = 25 technical replicates). Data are presented as mean ± SD. One-way ANOVA.
(F) Western blot analysis of cGAS-STING-TBK1 pathway activation in HCC4006, H1975, H358, and H1573 cells treated with olaparib, HER3-DXd, or their combination. 10 μg/mL HER3-DXd and 1 μM olaparib were used. Hsp90 was used as a loading control.
(G) cGAS/STING RNA-seq gene expression in HCC4006, H1975, H358, and H1573 cell lines, using publicly available RNA-seq data from the Cancer Cell Line Encyclopedia (CCLE) dataset. Expression values are shown as transcripts per million (TPM).
(H and I) Immunohistochemistry analysis of cGAS in paraffin-embedded HCC4006 and H1573 xenograft tissue; n = 2 from each condition. 3 mg/kg HER3-DXd, 100 mg/kg olaparib, or their combination was used.
(J and K) Heatmap comparing changes of cytokine expression in HCC4006/H358 cells co-cultured with NK cells between four conditions: control, olaparib treatment, HER3-DXd treatment, and their combination. *$p < 0.05$; **$p < 0.01$; ***$p < 0.001$; and ****$p < 0.0001$; ns, not significant.
See also Figure S5.

inhibition in preclinical models.[25] This suggests that HER3-DXd not only helps overcome resistance mechanisms to targeted therapies but could also modulate the tumor immune microenvironment to enhance the efficacy of immunotherapies. In our study, lung cancer cell lines treated with a combination of olaparib and HER3-DXd demonstrated enhanced activation of innate immune responses, in addition to a significant impact on tumor cell viability. These findings support the hypothesis that this combination therapy may amplify immune-mediated antitumor responses.

Other studies have similarly demonstrated the potential of HER3-targeting strategies in conjunction with immune-modulatory treatments. For example, treatment with a HER3 monoclonal antibody combined with osimertinib increased NK cell infiltration *in vivo* and activated the STING pathway *in vitro.*[26] Interestingly, we observed variability in the activation capacity of the STING response between cell lines. This variation may stem from differences in the expression or mutations in key components of the STING pathway or epigenetic silencing, which could impact the full signaling cascade. It is well documented that STING activity is hindered by epigenetic silencing such as promoter hypermethylation, and this can be reversed by demethylating agents or other epigenetic modulators.[27–29] Successful activation by STING agonists is also dependent on the promoter state. We also observed that the baseline cGAS expression, as well as the intactness of the STING downstream signaling, is critical for the innate immune effects induced by HER3-DXd and its combination with olaparib.

Regardless of their direct on-target effects, it is increasingly being recognized that ADCs, in general, can impact antitumor immunity and immune microenvironment. Besides innate immunity investigated in this study, a number of studies have shown that ADCs can indirectly enhance immune cell infiltration, secretion of immunomodulatory cytokines, and memory formation. Combinations of ADCs with immune checkpoint inhibitors (ICIs), beyond HER3-DXd, have been actively investigated in studies, some of which have reached clinical trials[30].

In conclusion, our study underscores the potential of HER3-DXd in combination with olaparib as an effective therapeutic strategy for NSCLC. This combination not only inhibits tumor growth and enhances survival in preclinical models but also activates the STING pathway and potentiates immune responses. These results highlight the potential of HER3-DXd, when used with olaparib, to overcome resistance mechanisms and modulate the tumor immune microenvironment. The observed variations in STING activation across different cell lines emphasize the complexity of tumor immune evasion mechanisms and warrant further investigation to uncover the regulatory factors involved. Ultimately, these findings support the clinical exploration of this combination therapy, particularly for tumors with HER3 expression, and stress the importance of targeting both tumor cell proliferation and immune modulation for better therapeutic outcomes in NSCLC.

Limitations of the study

The use of a restricted number of organoid lines and the immunocompromised mice in the *in vivo* study limits the generalizability of the results, and the immunogenic effects should be studied more broadly in a larger number of immunocompetent models. The NK cell experiments were conducted with cells from buffy coats derived from healthy donors, and they might behave differently from patients' own NK cells within the tumor microenvironment, which have been co-evolving with the tumor and might be suppressed by the tumor microenvironment. The potential toxicities of the identified drug combination has to be addressed in more detail before moving to human trials.

Although the combination treatment demonstrated promising antitumor activity and was generally tolerated under the conditions tested, the safety profile requires further characterization before clinical translation. While no significant hematological toxicities were observed, a late increase in serum ALT levels was detected in mice receiving the combination treatment, suggesting a potential hepatic effect associated with prolonged exposure. Dedicated toxicology studies are, therefore, warranted to define the therapeutic window, characterize potential organ-specific toxicities, and establish appropriate monitoring and dose-management strategies. If future clinical studies confirm a hepatic toxicity signal, liver function monitoring and treatment modifications, such as dose interruptions or drug holidays, may be required to optimize the balance between efficacy and tolerability.

RESOURCE AVAILABILITY

Lead contact

For additional information and resources, please contact the lead author, Heidi M. Haikala (heidi.haikala@helsinki.fi).

Materials availability

This study did not generate new, unique reagents. Laboratory-generated reagents used in this study were previously described[31] and are available from the lead contact upon reasonable request, with a completed material(s) transfer agreement.

Data and code availability

- Non-sensitive data reported in this paper will be shared by the lead contact upon request for research purposes.
- This paper analyzes existing, publicly available data at the Broad Institute CCLE at https://portals.broadinstitute.org/ccle. No new sequencing datasets were generated as part of this study.
- This paper does not report original code.
- Any additional information required to reanalyze the data reported in this paper is available from the lead contact upon request.

ACKNOWLEDGMENTS

This work was funded by the National Cancer Institute grant R35CA220497 (to P.A.J.). H.M.H was funded by the Research Council of Finland, the Sigrid Jusélius Foundation, the Jane and Aatos Erkko Foundation, the Cancer Foundation Finland, the Finnish Research Impact Foundation, the Business Finland, the Nummela Foundation, the Tampereen Tuberkuloosisäätiö, the Instrumentarium Science Foundation, and the Finnish Cultural Foundation. I.A.L was funded by the Swedish Cultural Foundation and Liv och Hälsa r.f. Support for experimental costs was provided by Daiichi Sankyo. Imaging was performed at the Biomedicum Imaging Unit, the University of Helsinki, supported by the Helsinki Institute of Life Science (HiLIFE) and Biocenter Finland. Histopathology services were performed at FIMM Digital Microscopy and Molecular Pathology Unit supported by HiLIFE and

Biocenter Finland.Open access was funded by Helsinki University Library. Schematics were created in BioRender by L. Lin and H.K.L. (2025/2026).

AUTHOR CONTRIBUTIONS

L. Lin, N.M., and I.A.L. contributed equally to the work by conducting experiments, analyzing data, and writing and editing the manuscript; L. Lahtinen., H.K.L., T.L., S.J., A.K., and K.M.S. conducted experiments and analyzed data; B.A. conducted experiments; I.I. provided funding and samples; P.C.G. provided funding, planned the study, analyzed data, and supervised the project; P.A.J. planned the study, provided funding, and supervised the project; and H.M.H. conducted experiments, analyzed data, provided funding, planned the study, and supervised the project.

DECLARATION OF INTERESTS

H.M.H. has been working within the past 5 years as a part-time Medical Advisor for Amgen AB and is a co-founder and shareholder of Solid Immuno-Oncology Oy. P.A.J has consulting fees from AstraZeneca, Boehringer-Ingelheim, Pfizer, Roche/Genentech, Takeda Oncology, ACEA Biosciences, Eli Lilly and Company, Araxes Pharma, Ignyta, Mirati Therapeutics, Novartis, LOXO Oncology, Daiichi Sankyo, Sanofi Oncology, Voronoi, SFJ Pharmaceuticals, Takeda Oncology, Transcenta, Silicon Therapeutics, Syndax, Nuvalent, Bayer, Esai, Biocartis, Allorion Therapeutics, Accutar Biotech and Abbvie, Monte Rosa, Scorpion Therapeutics, Merus, Frontier Medicines, Hongyun Biotechnology, and Duality; post-marketing royalties from DFCI-owned intellectual property on *EGFR* mutations licensed to Lab Corp; sponsored research agreements with AstraZeneca, Daichi-Sankyo, PUMA, Boehringer Ingelheim, Eli Lilly and Company, Revolution Medicines, and Astellas Pharmaceuticals; and stock ownership in Gatekeeper Pharmaceuticals.

STAR★METHODS

Detailed methods are provided in the online version of this paper and include the following:

- KEY RESOURCES TABLE
- EXPERIMENTAL MODEL AND STUDY PARTICIPANT DETAILS
  - Animal models
  - Biological samples
  - Cell lines and cell culture
- METHOD DETAILS
  - Cell viability and combination efficacy assays
  - Western blot
  - Drug experiment with tumor-on-a-chip
  - Live/dead assay
  - Flow cytometry
  - Isolating and expanding NK cells from PBMCs
  - Co-culture experiment with NK cells
  - siRNA-mediated silencing of the cGAS/STING pathway
  - Cytokine profiling
  - Immunohistochemistry
- QUANTIFICATION AND STATISTICAL ANALYSIS
  - Image quantification
  - Gene expression analysis of STING pathway in cell lines
  - Statistical analysis

SUPPLEMENTAL INFORMATION

Supplementary data related to this article can be found online at https://doi.org/10.1016/j.xcrm.2026.103002.

Received: June 8, 2025
Revised: June 10, 2026
Accepted: July 27, 2026
Published: August 21, 2026

REFERENCES

1. Chen, Z., Fillmore, C.M., Hammerman, P.S., Kim, C.F., and Wong, K.K. (2014). Non-small-cell lung cancers: a heterogeneous set of diseases. Nat. Rev. Cancer *14*, 535–546. https://doi.org/10.1038/nrc3775.
2. Ramalingam, S.S., Vansteenkiste, J., Planchard, D., Cho, B.C., Gray, J.E., Ohe, Y., Zhou, C., Reungwetwattana, T., Cheng, Y., Chewaskulyong, B., et al. (2020). Overall Survival with Osimertinib in Untreated, *EGFR*-Mutated Advanced NSCLC. N. Engl. J. Med. *382*, 41–50. https://doi.org/10.1056/NEJMoa1913662.
3. de Langen, A.J., Johnson, M.L., Mazieres, J., Dingemans, A.-M.C., Mountzios, G., Pless, M., Wolf, J., Schuler, M., Lena, H., Skoulidis, F., et al. (2023). Sotorasib versus docetaxel for previously treated non-small-cell lung cancer with KRAS^{G12C} mutation: a randomised, open-label, phase 3 trial. Lancet *401*, 733–746. https://doi.org/10.1016/S0140-6736(23)00221-0.
4. Liu, J., Kang, R., and Tang, D. (2022). The KRAS-G12C inhibitor: activity and resistance. Cancer Gene Ther. *29*, 875–878. https://doi.org/10.1038/s41417-021-00383-9.
5. Ocana, A., Vera-Badillo, F., Seruga, B., Templeton, A., Pandiella, A., and Amir, E. (2013). HER3 overexpression and survival in solid tumors: a meta-analysis. J. Natl. Cancer Inst. *105*, 266–273. https://doi.org/10.1093/jnci/djs501.
6. Berghoff, A.S., Magerle, M., Ilhan-Mutlu, A., Dinhof, C., Widhalm, G., Dieckman, K., Marosi, C., Wöhrer, A., Hackl, M., Zöchbauer-Müller, S., et al. (2013). Frequent overexpression of ErbB–receptor family members in brain metastases of non-small cell lung cancer patients. APMIS *121*, 1144–1152. https://doi.org/10.1111/apm.12063.
7. Scharpenseel, H., Hanssen, A., Loges, S., Mohme, M., Bernreuther, C., Peine, S., Lamszus, K., Goy, Y., Petersen, C., Westphal, M., et al. (2019). EGFR and HER3 expression in circulating tumor cells and tumor tissue from non-small cell lung cancer patients. Sci. Rep. *9*, 7406. https://doi.org/10.1038/s41598-019-43678-6.
8. Chen, Q., Jia, G., Zhang, X., and Ma, W. (2023). Targeting HER3 to overcome EGFR TKI resistance in NSCLC. Front. Immunol. *14*, 1332057. https://doi.org/10.3389/fimmu.2023.1332057.
9. Haikala, H.M., and Jänne, P.A. (2021). Thirty Years of HER3: From Basic Biology to Therapeutic Interventions. Clin. Cancer Res. *27*, 3528–3539. https://doi.org/10.1158/1078-0432.CCR-20-4465.
10. Hashimoto, Y., Koyama, K., Kamai, Y., Hirotani, K., Ogitani, Y., Zembutsu, A., Abe, M., Kaneda, Y., Maeda, N., Shiose, Y., et al. (2019). A Novel HER3-Targeting Antibody-Drug Conjugate, U3-1402, Exhibits Potent Therapeutic Efficacy through the Delivery of Cytotoxic Payload by Efficient Internalization. Clin. Cancer Res. *25*, 7151–7161. https://doi.org/10.1158/1078-0432.CCR-19-1745.
11. Yu, H.A., Goto, Y., Hayashi, H., Felip, E., Chih-Hsin Yang, J., Reck, M., Yoh, K., Lee, S.H., Paz-Ares, L., Besse, B., et al. (2023). HERTHENA-Lung01, a Phase II Trial of Patritumab Deruxtecan (HER3-DXd) in Epidermal Growth Factor Receptor-Mutated Non-Small-Cell Lung Cancer After Epidermal Growth Factor Receptor Tyrosine Kinase Inhibitor Therapy and Platinum-Based Chemotherapy. J. Clin. Oncol. *41*, 5363–5375. https://doi.org/10.1200/JCO.23.01476.
12. Haikala, H.M., Lopez, T., Köhler, J., Eser, P.O., Xu, M., Zeng, Q., Teceno, T.J., Ngo, K., Zhao, Y., Ivanova, E.V., et al. (2022). EGFR Inhibition Enhances the Cellular Uptake and Antitumor-Activity of the HER3 Antibody-Drug Conjugate HER3-DXd. Cancer Res. *82*, 130–141. https://doi.org/10.1158/0008-5472.CAN-21-2426.
13. Yonesaka, K., Takegawa, N., Watanabe, S., Haratani, K., Kawakami, H., Sakai, K., Chiba, Y., Maeda, N., Kagari, T., Hirotani, K., et al. (2019). An HER3-targeting antibody-drug conjugate incorporating a DNA topoisomerase I inhibitor U3-1402 conquers EGFR tyrosine kinase inhibitor-resistant NSCLC. Oncogene *38*, 1398–1409. https://doi.org/10.1038/s41388-018-0517-4.

CellPress
OPEN ACCESS

14. Flynn, P., Suryaprakash, S., Grossman, D., Panier, V., and Wu, J. (2024). The antibody-drug conjugate landscape. Nat. Rev. Drug Discov. *23*, 577–578. https://doi.org/10.1038/d41573-024-00064-w.

15. Fu, Z., Li, S., Han, S., Shi, C., and Zhang, Y. (2022). Antibody drug conjugate: the "biological missile" for targeted cancer therapy. Signal Transduct. Targeted Ther. *7*, 93. https://doi.org/10.1038/s41392-022-00947-7.

16. Staudacher, A.H., and Brown, M.P. (2017). Antibody drug conjugates and bystander killing: is antigen-dependent internalisation required? Br. J. Cancer *117*, 1736–1742. https://doi.org/10.1038/bjc.2017.367.

17. Samson, N., and Ablasser, A. (2022). The cGAS-STING pathway and cancer. Nat. Cancer *3*, 1452–1463. https://doi.org/10.1038/s43018-022-00468-w.

18. Ghandi, M., Huang, F.W., Jané-Valbuena, J., Kryukov, G.V., Lo, C.C., McDonald, E.R., 3rd, Barretina, J., Gelfand, E.T., Bielski, C.M., Li, H., et al. (2019). Next-generation characterization of the Cancer Cell Line Encyclopedia. Nature *569*, 503–508. https://doi.org/10.1038/s41586-019-1186-3.

19. Li, H., Ning, S., Ghandi, M., Kryukov, G.V., Gopal, S., Deik, A., Souza, A., Pierce, K., Keskula, P., Hernandez, D., et al. (2019). The landscape of cancer cell line metabolism. Nat. Med. *25*, 850–860. https://doi.org/10.1038/s41591-019-0404-8.

20. Barber, G.N. (2015). STING: infection, inflammation and cancer. Nat. Rev. Immunol. *15*, 760–770. https://doi.org/10.1038/nri3921.

21. Yu, H.A., Baik, C., Kim, D.W., Johnson, M.L., Hayashi, H., Nishio, M., Yang, J.C.H., Su, W.C., Gold, K.A., Koczywas, M., et al. (2024). Translational insights and overall survival in the U31402-A-U102 study of patritumab deruxtecan (HER3-DXd) in EGFR-mutated NSCLC. Ann. Oncol. *35*, 437–447. https://doi.org/10.1016/j.annonc.2024.02.003.

22. Yonesaka, K., Tanizaki, J., Maenishi, O., Haratani, K., Kawakami, H., Tanaka, K., Hayashi, H., Sakai, K., Chiba, Y., Tsuya, A., et al. (2022). HER3 Augmentation via Blockade of EGFR/AKT Signaling Enhances Anticancer Activity of HER3-Targeting Patritumab Deruxtecan in EGFR-Mutated Non-Small Cell Lung Cancer. Clin. Cancer Res. *28*, 390–403. https://doi.org/10.1158/1078-0432.CCR-21-3359.

23. Brasó-Maristany, F., Ferrero-Cafiero, J.M., Falato, C., Martínez-Sáez, O., Cejalvo, J.M., Margelí, M., Tolosa, P., Salvador-Bofill, F.J., Cruz, J., González-Farré, B., et al. (2024). Patritumab deruxtecan in HER2-negative breast cancer: part B results of the window-of-opportunity SOLTI-1805 TOT-HER3 trial and biological determinants of early response. Nat. Commun. *15*, 5826. https://doi.org/10.1038/s41467-024-50056-y.

24. Thomas, A., and Pommier, Y. (2019). Targeting Topoisomerase I in the Era of Precision Medicine. Clin. Cancer Res. *25*, 6581–6589. https://doi.org/10.1158/1078-0432.CCR-19-1089.

25. Haratani, K., Yonesaka, K., Takamura, S., Maenishi, O., Kato, R., Takegawa, N., Kawakami, H., Tanaka, K., Hayashi, H., Takeda, M., et al. (2020). U3-1402 sensitizes HER3-expressing tumors to PD-1 blockade by immune activation. J. Clin. Investig. *130*, 374–388. https://doi.org/10.1172/JCI126598.

26. Vicencio, J.M., Evans, R., Green, R., An, Z., Deng, J., Treacy, C., Mustapha, R., Monypenny, J., Costoya, C., Lawler, K., et al. (2022). Osimertinib and anti-HER3 combination therapy engages immune dependent tumor toxicity via STING activation in trans. Cell Death Dis. *13*, 274. https://doi.org/10.1038/s41419-022-04701-3.

27. Kitajima, S., Ivanova, E., Guo, S., Yoshida, R., Campisi, M., Sundararaman, S.K., Tange, S., Mitsuishi, Y., Thai, T.C., Masuda, S., et al. (2019). Suppression of STING Associated with LKB1 Loss in KRAS-Driven Lung Cancer. Cancer Discov. *9*, 34–45. https://doi.org/10.1158/2159-8290.CD-18-0689.

28. Falahat, R., Berglund, A., Perez-Villarroel, P., Putney, R.M., Hamaidi, I., Kim, S., Pilon-Thomas, S., Barber, G.N., and Mulé, J.J. (2023). Epigenetic state determines the in vivo efficacy of STING agonist therapy. Nat. Commun. *14*, 1573. https://doi.org/10.1038/s41467-023-37217-1.

29. Low, J.T., Chandramohan, V., Bowie, M.L., Brown, M.C., Waitkus, M.S., Briley, A., Stevenson, K., Fuller, R., Reitman, Z.J., Muscat, A.M., et al. (2022). Epigenetic STING silencing is developmentally conserved in gliomas and can be rescued by methyltransferase inhibition. Cancer Cell *40*, 439–440. https://doi.org/10.1016/j.ccell.2022.04.009.

30. Shi, R., Jia, L., Lv, Z., and Cui, J. (2025). Another power of antibody-drug conjugates: immunomodulatory effect and clinical applications. Front. Immunol. *16*, 1632705. https://doi.org/10.3389/fimmu.2025.1632705.

31. Alsaed, B., Smolander, J., Laitinen, H., Lin, L., Bobik, N., Lahtinen, L., Räsänen, M., Jansouz, S., Peltonen, K., Jokinen, E., et al. (2024). Ex vivo modeling of precision immuno-oncology responses in lung cancer. Sci. Adv. *10*, eadq6830. https://doi.org/10.1126/sciadv.adq6830.

32. Bankhead, P., Loughrey, M.B., Fernández, J.A., Dombrowski, Y., McArt, D.G., Dunne, P.D., McQuaid, S., Gray, R.T., Murray, L.J., Coleman, H.G., et al. (2017). QuPath: Open source software for digital pathology image analysis. Sci. Rep. *7*, 16878. https://doi.org/10.1038/s41598-017-17204-5.

33. Kurppa, K.J., Liu, Y., To, C., Zhang, T., Fan, M., Vajdi, A., Knelson, E.H., Xie, Y., Lim, K., Cejas, P., et al. (2020). Treatment-Induced Tumor Dormancy through YAP-Mediated Transcriptional Reprogramming of the Apoptotic Pathway. Cancer Cell *37*, 104–122.e12. https://doi.org/10.1016/j.ccell.2019.12.006.

STAR★METHODS

KEY RESOURCES TABLE

REAGENT or RESOURCE	SOURCE	IDENTIFIER
Antibodies		
HER3/ErbB3	Cell Signaling	Cat#12708; RRID: AB_2721919
APC anti-human erbB3/HER-3 antibody	BioLegend	Cat#342708
ATM	Cell Signaling	Cat#2873; RRID: AB_2061659
pATM (Ser1981)	Cell Signaling	Cat#13050; RRID: AB_2789100
pATM-PE	BioLegend	Cat#651203; RRID: AB_2562654
p21	Cell Signaling	Cat#2947; RRID: AB_823586
pH2AX (Ser139)	Cell Signaling	Cat#80312; RRID: AB_2799949
pH2AX (Ser139), FITC	Sigma Aldrich	Cat#16-202A; RRID: AB_568825
Propidium Iodide	Fisher Scientific	Cat#11599296
cGAS	Invitrogen	Cat#PA5-141097; RRID: AB_2932549
STING (D2P2F)	Cell Signaling	Cat#13647; RRID: AB_2732796
TBK1/NAK	Cell Signaling	Cat#3013; RRID: AB_2199749
pTBK1 (Ser172)	Invitrogen	Cat#PA5-105919; RRID: AB_2817318
Hsp90 (C45G5)	Cell Signaling	Cat#4877; RRID: AB_2233307
Goat anti-Mouse IgG (H + L) Secondary Antibody, HRP	Invitrogen	Cat#31430; RRID: AB_228307
Goat anti-Rabbit IgG (H + L) Secondary Antibody [HRP]	Novus Biologicals	Cat#NB7160; RRID: AB_10124655
Biological samples		
Buffy coat from whole blood	Finnish Red Blood Bank	N/A
Chemicals, peptides, and recombinant proteins		
HER3-DXd	Provided by Daiichi Sankyo	N/A
Olaparib	FIMM, HiLIFE, University of Helsinki	N/A
DXd	MedChemExpress	Cat#HY-13631D
Advanced DMEM/F-12	Gibco	Cat#12634028
GlutaMAX™ Supplement	Gibco	Cat#35050061
Penicillin-Streptomycin (10,000 U/mL)	Gibco	Cat#15140122
B-27™ Supplement (50×), serum free	Gibco	Cat#17504044
HEPES (1M)	Gibco	Cat#15630056
Human R-Spondin 1 Recombinant Protein	PeproTech	Cat#120-38
Nicotinamide	SigmaAldrich	Cat#N0636-100G
N-acetyl-L-cysteine	SigmaAldrich	Cat#A9165-5G
Primocin	Invivogen	Cat#ant-pm-05
Human Noggin Recombinant Protein	PeproTech	Cat#120-10C
Human FGF-10 Recombinant Protein	PeproTech	Cat#100-26
Human FGF-7 Recombinant Protein	PeproTech	Cat#100-19
Y-27632 dihydrochloride	Selleckchem	Cat#S1049
A83-01	SigmaAldrich	Cat#SML0788-5MG
SB202190	MedChemExpress	Cat#HY-10295
NK MACS® Medium, human	Miltenyi Biotec	Cat#130-114-429
Human IL-2, Animal-Free Recombinant Protein	PeproTech	Cat#AF-200-02
Human IL-15 Recombinant Protein	PeproTech	Cat#200-15
Matrigel® Growth Factor Reduced (GFR) Basement Membrane Matrix	Corning	356231

(*Continued on next page*)

Continued		
REAGENT or RESOURCE	SOURCE	IDENTIFIER
TrypLE™ Express Enzyme (1×), no phenol red	Gibco	Cat#12604-054
Sodium bicarbonate	SigmaAldrich	Cat#S5761
Cultrex 3-D Culture Matrix Rat Collagen I	BioTechne	Cat#3447-020-01
Fibronectin	Roche	Cat#1083803900
Endothelial Cell Growth Medium 2	PromoCell	Cat#C-22011
Trypsin-EDTA 0.05%	Gibco	Cat#25300062
ViaStain AOPI Staining Solution	Revvity	Cat#CS2-0106
RIPA Lysis and Extraction Buffer	ThermoScientific	Cat#89900
Halt™ Protease Inhibitor Single-Use Cocktail	ThermoScientific	Cat#78430
Halt™ Phosphatase Inhibitor Single-Use Cocktail	ThermoScientific	Cat#78428
PageRuler™ Prestained Protein Ladder, 10 to 180 kDa	ThermoScientific	Cat#26617
PageRuler™ Plus Prestained Protein Ladder, 10 to 250 kDa	ThermoScientific	Cat#26620
5× siRNA Buffer	Horizon Discovery	B-002000-UB-100
DharmaFECT 1 Transfection Reagent	Horizon Discovery	T-2001-01
Critical commercial assays		
CellTiter-Glo® Luminescent Cell Viability Assay	Promega	Cat#G7571
MycoAlert® PLUS Mycoplasma Detection Kit	Lonza	Cat#LT07-710
CellEvent™ Caspase-3/7 Green ReadyProbes™ Reagent	R37111	Cat#R37111
NK Cell Isolation Kit, human	Miltenyi Biotec	Cat#130-092-657
CellTox™ Green Cytotoxicity Assay	Promega	Cat#G8731
Bio-Plex Pro Human Immunotherapy Panel, 20-Plex	BioRad	Cat#12007975
Deposited data		
CCLE_RNAseq_rsem_genes_tpm_20180929.txt.gz	Broad Institute Cancer Cell Line Encyclopedia (CCLE)	https://data.broadinstitute.org/ccle/CCLE_RNAseq_rsem_genes_tpm_20180929.txt.gz
Experimental models: Cell lines		
HCC4006; human EGFR-mutant NSCLC, male	ATCC	(CRL-2871); RRID: CVCL_1269
H1975; human EGFR-mutant NSCLC, female	ATCC	(CRL-5908); RRID: CVCL_1511
DFCI243; human EGFR-mutant NSCLC, female	Established in the Jänne laboratory	N/A
DFCI284; human EGFR-mutant NSCLC	Established in the Jänne laboratory	N/A
PC-9; human EGFR-mutant NSCLC, male	Dr. Kazuto Nishio (Kindai University, Osaka, Japan)	Fingerprinted; RRID: CVCL_B260
H1373; human KRAS-mutant NSCLC, male	ATCC	CRL-5866; RRID:CVCL_1465
H1573; human KRAS-mutant NSCLC, female	ATCC	CRL-5877; RRID:CVCL_1478
H358; human KRAS-mutant NSCLC, male	ATCC	CRL-5807; RRID:CVCL_1559
LCLC-97TM1; human KRAS-mutant LCLC, male	CLS/Cytion	RRID:CVCL_1376
A549; human KRAS-mutant NSCLC, male	ATCC	CCL-185; RRID:CVCL_0023
H23; human KRAS-mutant NSCLC, male	FIMM, HiLIFE, University of Helsinki	RRID:CVCL_1547
H2228; human ALK + HGNC- gene fusion NSCLC, Female	Dr. Anna-Liisa Levonen-Harju (University of Eastern Finland, Kuopio, Finland)	RRID:CVCL_1543
ORG29; human EGFR-mutant NSCLC, female	Established in HaikaLab	N/A
LUNG31; human EGFR-mutant NSCLC, female	Established in HaikaLab	N/A
ORG54: human KRAS-mutant NSCLC, female	Established in HaikaLab	N/A
Experimental models: Organisms/strains		
Mouse: NOD.Cg-Prkdc^{scid} Il2rg$^{tm1Wjl/SzJ}$ (NSG)	The Jackson Laboratory	Strain#:005557

(*Continued on next page*)

Continued		
REAGENT or RESOURCE	SOURCE	IDENTIFIER
Oligonucleotides		
ON-TARGETplus Non-targeting Control siRNA #1	Horizon Discovery	D-001810-01-05
ON-TARGETplus siRNA TMEM173 human, SMARTPOOL	Horizon Discovery	L-024333-00-0005
ON-TARGETplus siRNA MB21D1 human, SMARTPOOL	Horizon Discovery	L-015607-02-0005
Software and algorithms		
FlowJo version 10.9 (TreeStar)	FlowJo	https://flowjo.com/flowjo/overview
ImageJ, version 1.54g	ImageJ	https://imagej.net/ij/download.html
Python 3.13.2.	Python	https://www.python.org/downloads/release/python-3132/
Graphpad Prism, version 10.2.2	Graphpad	https://graphpad.com
QuPath (0.6.0-arm64)	Bankhead et al.[32]	https://qupath.github.io/

EXPERIMENTAL MODEL AND STUDY PARTICIPANT DETAILS

Animal models

Cell-line derived xenograft studies: Female NSG (NOD.Cg-Prkdc^{scid} Il2rg^{tm1Wjl}/SzJ) mice, 6-weeks old were purchased from the Jackson laboratories. Five million HCC4006 or 3.5 million H1573 cells in 50% matrigel-PBS were injected subcutaneously in the right flank of the NSG mice, and tumor volumes and mice weights were recorded bi-weekly. When the tumors reached an average size of 150–175 mm^3, mice were randomized into various treatment groups as follows: vehicle control (10% DMSO, 50% of 60% of 2-hydroxypropyl-β-cyclodextrin (HPbCD) and 40% water, once daily by oral gavage), HER3-DXd (in 10 mM acetate buffer with 5% sorbitol, pH 5.5) at 3 mg/kg once weekly for 4 doses, olaparib (in 10% DMSO, 50% of 60% of HPbCD and 40% water) at 100 mg/kg once daily for 28 days by oral gavage or the combination of HER3-DXd and olaparib. Following completion of treatment, mice were followed for tumor re-growth and mice with tumor volumes exceeding 2000 mm^3 or if the tumor became ulcerated or necrotic, mice were euthanized. All breeding, animal husbandry, and *in vivo* experiments were approved by the Dana-Farber Cancer Institute Animal Care and Use Committee (IACUC; Protocol No. 04–111) and performed in accordance with institutional guidelines. Only female mice were used; therefore, sex-dependent differences were not assessed.

Biological samples

For patient-derived tumor organoids: A statement of approval for the study protocol was given by the Ethics Committee of the Hospital District of Helsinki and Uusimaa (HUS/970/2021) and research permit was granted by Helsinki University Hospital (HUS/334/2026). The organoid models were derived from tumor samples from three female patients: ORG-29 (65 years), LUNG-31 (57 years), and ORG-54 (78 years). For buffy coats: Whole blood from one healthy donor was obtained from the Finnish Red Cross Blood Service (permit number: 24/2025). For donor blood, information regarding sex/gender was not available and therefore was not considered in the analyses.

Cell lines and cell culture

The NSCLC cell lines HCC4006, H1975, PC-9, H1373, H1573, H358, A549, H23, H2228, the patient-derived DFCI cell lines and the large cell lung carcinoma LCLC-97TM1 and were cultured in RPMI1640 (Euroclone) with 10% FBS (SigmaAldrich), and 1% L-glutamine (Gibco) and penicillin/streptomycin (Euroclone). The patient-derived NSCLC ORG cell lines were grown in special organoid medium. Special organoid medium consists of advanced DMEM/F-12 (Gibco), GlutaMAX supplement (Gibco), penicillin/streptomycin (Gibco), B-27 supplement (Gibco), HEPES 1M (Gibco), R-Spondin 1 (PeproTech), nicotinamide (Merck), N-acetylcysteine (Merck), primocin (Invivogen), noggin (PeproTech), human recombinant FGF-10 (PeproTech), rho kinase inhibitor Y-27632 (Selleckchem), human recombinant FGF-7 (PeproTech), A83-01 (Merck), and SB202190 (p38 inhibitor) (MedChemExpress).

Human umbilical vein endothelial cells (HUVECs) were a kind gift from Prof. Saharinen's laboratory (University of Helsinki) and grown in endothelial cell growth medium 2 (PromoCell). PC-9 cells were a kind gift from Dr. Kazuto Nishio (Kindai University, Osaka, Japan). HCC4006, H1975, H1373, and H1573 cells were purchased from ATCC. H358, A549, and LCLC-97TM1 cells were purchased from CSL/Cytion. H23 cells were a kind gift from FIMM, HiLIFE, University of Helsinki, and H2228 cells were a kind gift from Dr. Anna-Liisa Levonen-Harju (University of Eastern Finland, Kuopio, Finland). DFCI cell lines were established as previously described.[33] ORG cell lines were established from patient-derived tumors as previously described.[31] The following cell lines have been fingerprinted: HCC4006, H1975, and PC-9. Mycoplasma was routinely tested with MycoAlert PLUS Mycoplasma Detection Kit (Lonza) and if necessary, treated with plasmocin (Invivogen). All cells were cultured in +37°C, 5% CO_2.

HER3-DXd was provided by Daiichi Sankyo, DDR inhibitors were a kind gift from Prof. David Barbie's laboratory (DFCI & Harvard). Olaparib was purchased from Biomedicine unit at FIMM, University of Helsinki.

METHOD DETAILS

Cell viability and combination efficacy assays

For IC50 assays, 500 cells per well were seeded per well on 384-well plates, and on the following day drugs were added by using HP300e digital dispenser (HP). After 72 h of drug treatment, cell viability was assayed by using CellTiter Glo (Promega) according to the manufacturer's instructions. For cell growth and combination efficacy assays, 1000 cells per well were seeded on a 96-well plate, and drugs were added the following day by using HP300e digital dispenser. Cell confluency was followed up by using IncuCyte S3 live cell analysis system (Sartorius). CellEvent Caspase-3/7 Green Ready Probes (Fisher Scientific) were used for apoptosis assays. DDR inhibitors were a kind gift from Prof. David Barbie's laboratory (DFCI & Harvard).

Western blot

Cells were plated at a density of 0.3×10^6 per well in a 6-well plate, and if pertinent, treated the next day. At specific timepoints, the cells were lysed in RIPA buffer (ThermoScientific) supplemented with Halt Protease and/or Phosphatase inhibitor cocktail (ThermoScientific). Protein concentration of the samples was determined using the Pierce BCA Protein Assay Kit (ThermoScientific), following the manufacturer's protocol. 30 μg of total protein was separated with SDS-PAGE, followed by transfer using the Trans-Blot Turbo RTA Transfer Kit (BioRad) and Trans-Blot Turbo Transfer System (Bio-Rad). Primary antibodies were diluted to 1:1000 and secondary antibodies were diluted to 1:10 000 for immunoblotting. HER3 was immunoblotted with HER3/ErbB3 (Cell Signaling, Cat# 12708), DNA damage response biomarkers with pATM (Ser1981) (Cell Signaling, Cat# 13050), p21 (Cell Signaling, Cat# 2947) and pH2aX (Ser139) (Cell Signaling, Cat# 80312), and cGAS-STING pathway with the following antibodies: cGAS (Invitrogen, Cat# PA5-141097), STING (D2P2F) (Cell Signaling, Cat# 13647), TBK1/NAK (Cell Signaling, Cat# 3010) and pTBK1 (Ser172) (Invitrogen, Cat# PA5-105919). Hsp90 (C45G5) (Cell Signaling, Cat# 4877) was used as a loading control for all the blots, either goat anti-mouse or goat anti-rabbit IgG secondary antibody (Invitrogen, Cat# 31430; Novus Biologicals, Cat# NB7160) was used as secondary antibody. Prestained protein ladder PageRuler (ThermoScientific) was used as a molecular weight marker. Detection was performed using Clarity Western ECL Substrate (Bio-Rad), and membranes were visualized with NIR fluorescent imager (Azure Biosystems).

Drug experiment with tumor-on-a-chip

Patient-derived tumor organoids were dissociated from Matrigel (Corning) with pre-warmed TrypLE Express Enzyme (Gibco) and incubated at +37°C for 5–10 min, until they were single cells or smaller aggregates. The enzymatic process was stopped by washing the cells twice with cold DMEM (EuroClone) for 5 min, 300g at +4°C. The cells were resuspended in 1 mL special organoid medium, counted, and to have at least 7000 cells per idenTx microfluidic device (IDTX40, Aim Biotech), 250 000 cells were transferred to another tube. A collagen mixture was prepared on ice with 37g/L sodium bicarbonate ($NaHNO_3$) (reconstituted in water, pH 9.5) (SigmaAldrich), HEPES (Gibco) and Rat Collagen I (BioTechne) in 1:1:8 ratiorespectively. $NaHNO_3$ and HEPES were mixed thoroughly first before adding Collagen I. The 250 000 cells were centrifuged for 5 min, 300g at +4°C, resuspended with 400 μL of the collagen mixture, then 5 μL of cell resuspension was carefully loaded into each middle channel of the 3-lane idenTx chip, incubated for 30 min, +37°C, 5% CO_2.

After the solidification of the collagen had been assessed with a Nikon Eclipse TS100 microscope, the upper channel was coated with 10 μL of 50 μg/mL Fibronectin (Roche) in PBS, and 100 μL of previously special organoid media was added to the lower media channel. The media flow was checked before incubating for 1 h, +37°C, 5% CO_2.

HUVECs were detached with pre-warmed Trypsin-EDTA 0.05% (Gibco), washed with warm DMEM for 5 min 300g, resuspended in pre-warmed endothelial cell growth medium 2, then counted. To have 15 000 HUVECs/chip, the cells were diluted to 3000 cells/μL. After the fibronectin was washed out of all the upper channels with RT PBS 3×, 5 μL of the HUVEC cell suspension was loaded onto each upper channel, then incubated for 1–2 h, +37°C, 5% CO_2. After confirming the formation of the barrier and the condition of the collagen, unattached HUVECs were flushed with endothelial cell growth medium 2, then added 70–80 μL of said medium to each upper channel. Special organoid medium was added to lower media channels.

After confirming the media flow in all the channels, 50 μL PBS was added to the middle chamber, so the plate does not dry out. The plate was put on a rocker inside the incubator the next day at a speed of 1 rpm to create media flow. The formation of the tumor organoids was monitored every day, and every other day all the media was changed. After adequate organoid formation, the media from upper channels were removed, and drug treatments prepared in endothelial cell growth medium 2 were added 100 μL per upper channel.

Live/dead assay

The viability of tumor organoids in identX microfluidic devices was visualized with AO/PI staining (Revvity). All media channels were flushed and washed with PBS twice, 50 μL of diluted AO/PI (1:6 in PBS) was added into each media channel, then the plate was on a rocker for 15 min in +37°C, 5% CO_2. The stain was washed off with PBS twice, then added to the media channels to prevent the collagen from drying out. The viability was imaged using Nikon Eclipse Ti-E microscope (Biomedicum Imaging Unit, BIU), then quantified using ImageJ (1.54g).

Flow cytometry

Cells were detached with TrypLE Express (Gibco), both medium and cells were collected for flow cytometry analysis. Cells were stained with Trypan Blue (Invitrogen) and counted with Countess 3 FL Automated Cell Counter (Invitrogen), 200 000 cells were fixed with 4% PFA (Fisher Scientific) and permeabilized with 0.1% Triton X-100 in PBS (Sigma-Aldrich; Corning) was performed on cells in flow buffer (10% FBS in PBS) for 15–30 min in dark, whereafter cells were washed three times with cold PBS. BD FACSVerse cell analyzer (BD) was used for acquisition, and data was analyzed using FlowJo version 10.9 (TreeStar).

Isolating and expanding NK cells from PBMCs

Human peripheral blood mononuclear cells (PBMCs) were isolated from buffy coat (Finnish Red Blood Bank) using Ficoll-Paque. Natural killer (NK) cells were isolated with NK Cell Isolation kit (Miltenyi Biotec) as suggested by manufacturers protocol, as well as the phenotype and purity analysis with flow cytometry. Following Miltenyi Biotec's protocol, the isolated NK cells were expanded for 14 days with NK MACS Medium (Miltenyi Biotec), supplemented with IL-2 (2300 U/mL) and IL-15 (100 U/mL) (Peprotech; Miltenyi Biotec), then frozen down.

Co-culture experiment with NK cells

NK cells were thawed and cultured for three days in NK MACS Medium supplemented with IL-2 (2300 U/mL) and IL-15 (100 U/mL). Tumor cells were treated 48 h with drugs before co-culturing NKcells with them in 1:1 tumor and NK cell media with 0.3× CellTox (Promega). NK cells were cultured for 24 h with tumor cells whereafter the viability was analyzed by using CellTiter Glo (Promega). Live imaging and analysis were performed using CellCyte X (Cytena). Imaging was done every 30 min for 4 h after NK cell addition, whereafter the imaging was continued every 6 h for 20 h.

siRNA-mediated silencing of the cGAS/STING pathway

H358 cells were seeded at a density of 3,000 cells per well in 384-well plates. Gene silencing of cGAS (MB21D1) or STING (TMEM173) was performed using ON-TARGETplus siRNAs (ON-TARGETplus siRNA MB21D1 human and ON-TARGETplus siRNA TMEM173 human, Horizon Discovery). A non-targeting siRNA (ON-TARGETplus Non-targeting Control siRNA, Horizon Discovery) was used as a control. Transfections were carried out using DharmaFECT 1 Transfection Reagent (Horizon Discovery) according to the manufacturer's instructions. After 24 h of incubation, cells were treated with either 10 μg/mL HER3-DXd, 1 μM olaparib, or their combination, and incubated for an additional 48 h. The culture medium was then refreshed, and NK cells were added at a 1:2 tumor-to-NK cell ratio. Following a further 24-h incubation, cell viability was assessed using the CellTiter-Glo assay (Promega). All incubations were conducted at 37°C in 5% CO_2.

Cytokine profiling

A multiplex immunoassay was performed to profile cytokines from media samples collected from the NK co-culture experiment. Human Immunotherapy 20-plex panel (BioRad) was used with Luminex 200 instrument.

Immunohistochemistry

Paraffin embedded lung cancer xenograft tissue was sectioned, stained, and scanned at the FIMM Digital Microscopy and Molecular Pathology Unit as a service. The sections were pre-treated using heat mediated antigen retrieval with Tris-EDTA, pH 9.0 for 1 h. The tissues were blocked in 5% BSA for 30 min at room temperature, washed with ddH2O and PBS, and then probed with a dilution of 1:200 cGAS antibody (Invitrogen, Cat# PA5-141097) for 30 min at RT. DAB was used as the chromogen. Tissues were counterstained with hematoxylin and mounted with DPX.

QUANTIFICATION AND STATISTICAL ANALYSIS

Image quantification

Microscopy images were quantified by ImageJ (1.54g) software. Images were opened and color channels were split to allow separate quantification, with the green channel representing live cells and the red channel representing dead cells. Each channel was auto-adjusted for brightness and contrast, then converted to 8-bit, resulting in monochrome images. A measuring mask was generated, and the upper threshold was set to 255 while the lower threshold was adjusted to approximately 50–60. The masked area of each channel was analyzed to quantify the live and dead regions. Live and dead values from the same chip were combined to obtain the total. The live/dead ratio was calculated as follows: (live value/total) × 100 to determine the percentage of live organoids, and (dead value/total) × 100 to determine the percentage of dead organoids. IHC stainings were quantified using the QuPath (0.6.0-arm64) software.[32]

Gene expression analysis of STING pathway in cell lines

Gene expression of the cGAS/STING pathway in cell lines was assessed using CCLE RNA-seq TPM data obtained from the Broad Institute (CCLE_RNAseq_rsem_genes_tpm_20180929;https://data.broadinstitute.org/ccle).

Data were extracted for 11 NSCLC cell lines. Expression levels were calculated as the median $\log_2$(TPM +1) for genes of interest within a previously defined cGAS–STING signature, including key components cGAS, STING1, TBK1, IRF3, CCL5, and CXCL10. All analyses were performed using Python 3.13.2.

Statistical analysis

Data were analyzed with GraphPad Prism, version 10.2.2. Statistical tests used for each experiment are indicated in the corresponding figure legends. Unless otherwise stated, statistical significance was defined as $p < 0.05$. Asterisks indicate the following significance levels: *$p < 0.05$; **$p < 0.01$; ***$p < 0.001$; ****$p < 0.0001$; ns, not significant.

Cell Reports Medicine, Volume *7*

Supplemental information

PARP inhibition enhances the antitumor activity of HER3-DXd in non-small cell lung cancer

Linh Lin, Narges Moradi, Iris A. Lähdeniemi, Hanna K. Laitinen, Bassel Alsaed, Lilja Lahtinen, Timothy Lopez, Shadi Jansouz, Aine Knott, Kara M. Soroko, Ilkka Ilonen, Prafulla C. Gokhale, Pasi A. Jänne, and Heidi M. Haikala

Supplemental PDF (Linh et al., Cell Reports Medicine)

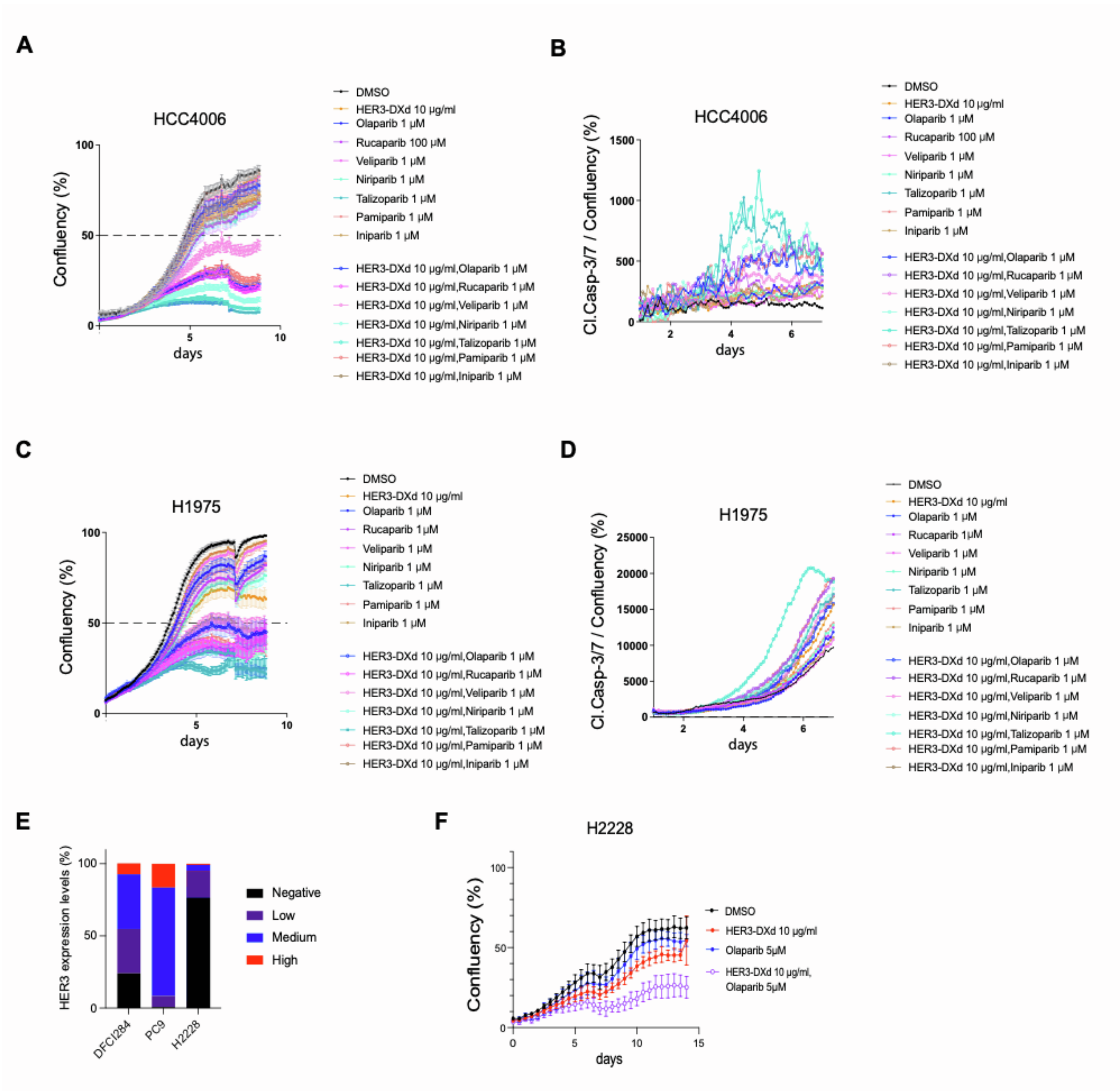

Figure S1: Additional data from EGFR-mutant cell lines (related to Figure 2). (A-D) Incucyte cell confluency and apoptosis assays from PARP inhibitor and HER3-DXd-treated cells over time. Data are representative of three individual experiments. (E) Flow cytometry quantification of HER-3 expression levels in DFCI284, PC9 and H2228, n = 3 biological experiments. (F) CellCyte cell confluency assay of H2228 treated with PARP inhibitor and HER3-DXd over time. Data are presented as mean ± SD. Data are representative of three individual experiments.

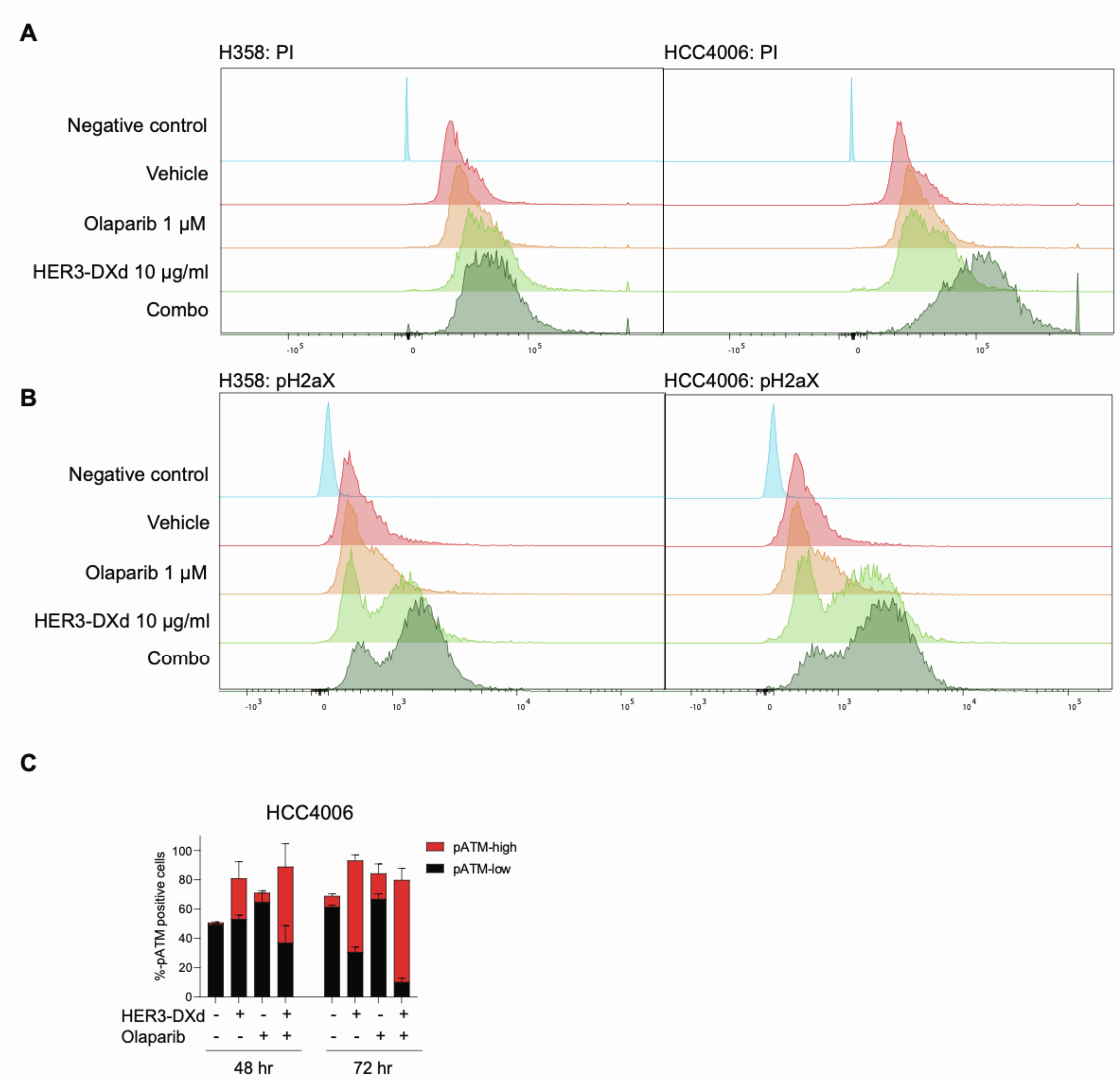

Figure S2: DNA damage response activation in H358 and HCC4006 (related to Figure 4). (A-B) Representative flow cytometry plots of PI and pH2aX staining in H358 and HCC4006 cells. (C) Quantification of pATM flow cytometry staining in HCC4006 cell line, n = 3 biological experiments. Data are presented as mean ± SD.

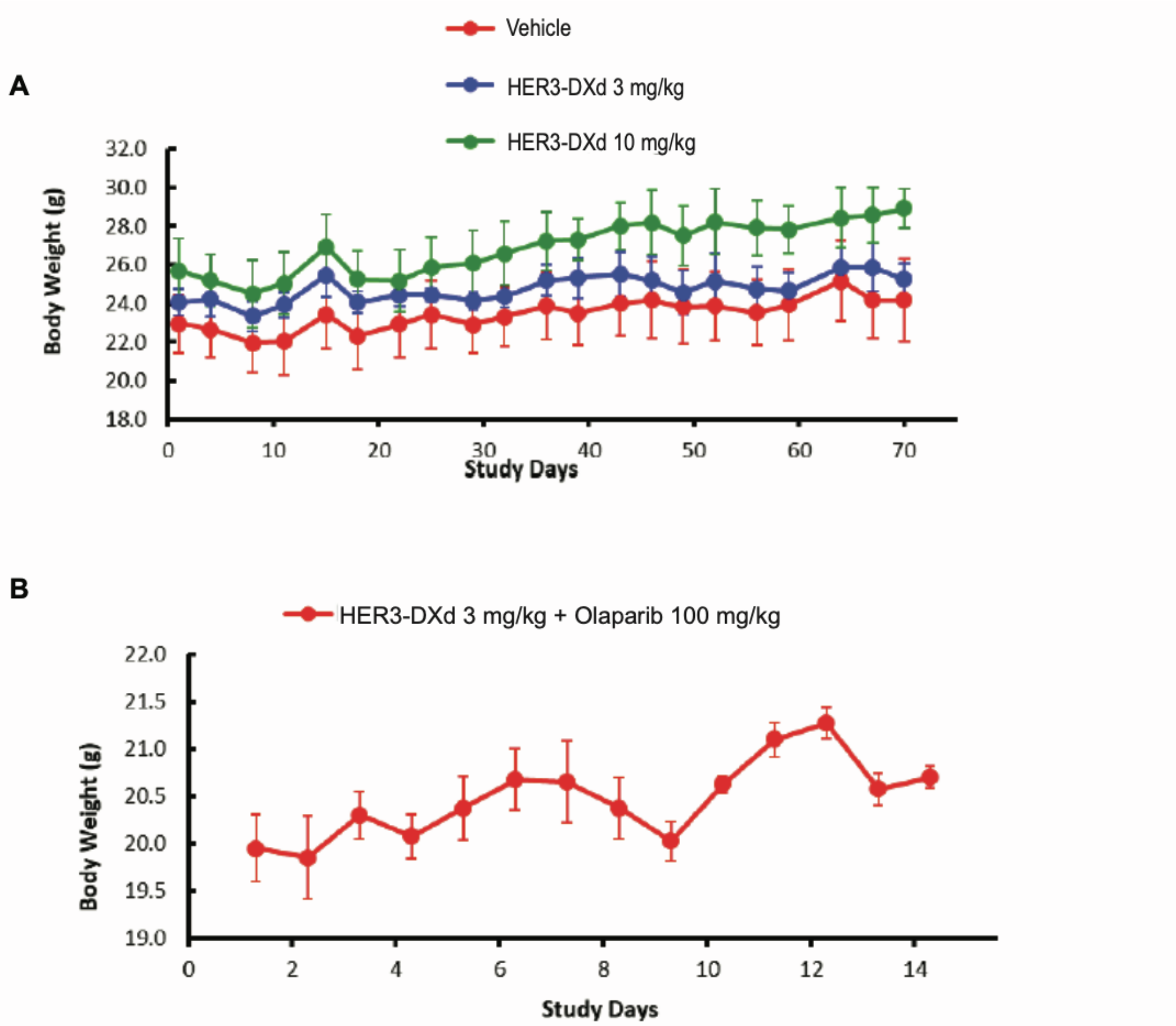

C

Pharmacodynamics Study Groups					
Group #	Test Agent	Dose	Dosing Volume	Dosing Schedule	Number of Animals
1	Vehicle	-	10 mL/kg	PO QD x 3 Days	3
2	HER3-DXd	3 mg/kg	10 mL/kg	IV x 1 Dose	3
3	Olaparib	100 mg/kg	10 mL/kg	PO QD x 3 Days	3
4	HER3-DXd + Olaparib	3 mg/kg + 100 mg/kg	10 mL/kg + 10 mL/kg	IV x 1 Dose + PO QD x 3 Days	3

Figure S3: ***In vivo*** **dose optimization and tolerability data in mice (related to Figure 5).** (A) Average body weight plot for pilot efficacy study represented as mean ± SEM. (B) Tolerability study with four female NSG mice weighed daily for two weeks represented as mean ± SEM. (C) Pharmacodynamics studies for the optimization of the drug dose and dosing strategies in mice.

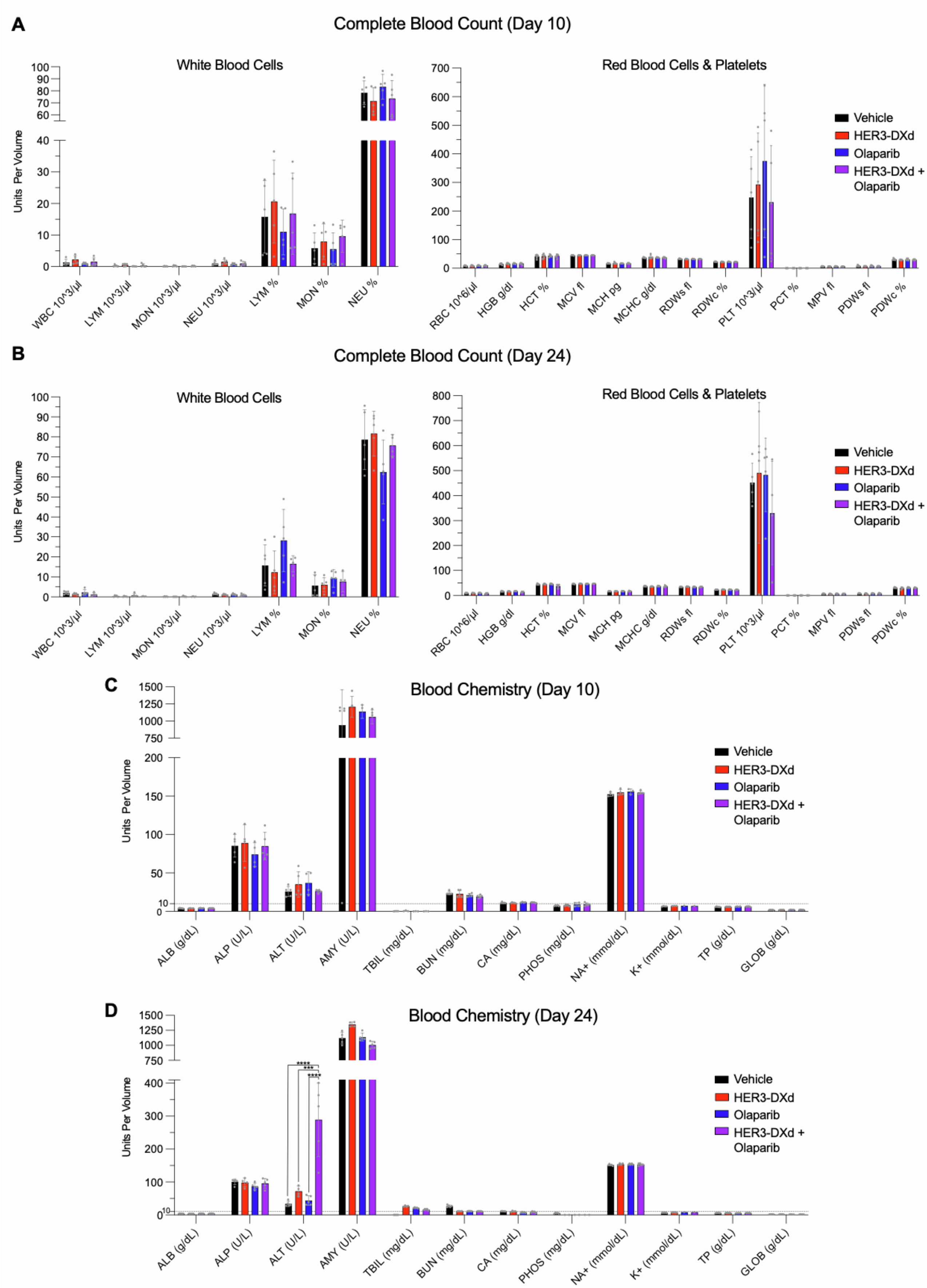
A
Complete Blood Count (Day 10)
White Blood Cells
Red Blood Cells & Platelets
Units Per Volume
Vehicle
HER3-DXd
Olaparib
HER3-DXd + Olaparib
WBC 10^3/µl
LYM 10^3/µl
MON 10^3/µl
NEU 10^3/µl
LYM %
MON %
NEU %
RBC 10^6/µl
HGB g/dl
HCT %
MCV fl
MCH pg
MCHC g/dl
RDWs fl
RDWc %
PLT 10^3/µl
PCT %
MPV fl
PDWs fl
PDWc %
B
Complete Blood Count (Day 24)
C
Blood Chemistry (Day 10)
D
Blood Chemistry (Day 24)
ALB (g/dL)
ALP (U/L)
ALT (U/L)
AMY (U/L)
TBIL (mg/dL)
BUN (mg/dL)
CA (mg/dL)
PHOS (mg/dL)
NA+ (mmol/dL)
K+ (mmol/dL)
TP (g/dL)
GLOB (g/dL)
****
***
****

Figure S4: ***In vivo* tolerability data of HER3-DXd in mice (related to Figure 5).**

(A) Complete blood count of mice on day 10. (B) Complete blood count of mice on day 24. (C) Blood chemistry of mice on day 10. (D) Blood chemistry of mice on day 24. Data are presented as mean ± SD, $n = 5$ mice per condition. One-way ANOVA. Treatments included HER3-DXd (3 mg/kg), olaparib (100 mg/kg), or their combination. WBC, white blood cells; NEU, neutrophils; LYM, lymphocytes; MON, monocytes; EOS, eosinophils; BAS, basophils; IMG, immature granulocytes; NEU%, percentage of neutrophils; LYM%, percentage of lymphocytes; MON%, percentage of monocytes; EOS%, percentage of eosinophils; BAS%, percentage of basophils; IMG%, percentage of immature granulocytes; RBC, red blood cells; HGB, hemoglobin; HCT, hematocrit; MCV, mean corpuscular volume; MCH, mean corpuscular hemoglobin; MCHC, mean corpuscular hemoglobin concentration; RDW-CV, coefficient of variation in red cell distribution width; RDW-SD, standard deviation in red cell distribution width; PLT, platelets; MPV, mean platelet volume; PDW, platelet distribution width; PCT, procalcitonin; P-LCC, platelet larger cell count; P-LCR, platelet larger cell ratio. ALB, albumin; ALP, alkaline phosphatase; ALT, alanine transaminase; AMY, amylase; TBIL, total bilirubin; PHOS, phosphorus; GLU, glucose; TP, total protein; GLOB, globulin.

Unless otherwise specified, results were not significant.

$P < 0.05$; ** $P < 0.01$; *** $P < 0.001$; **** $P < 0.0001$; ns, not significant.

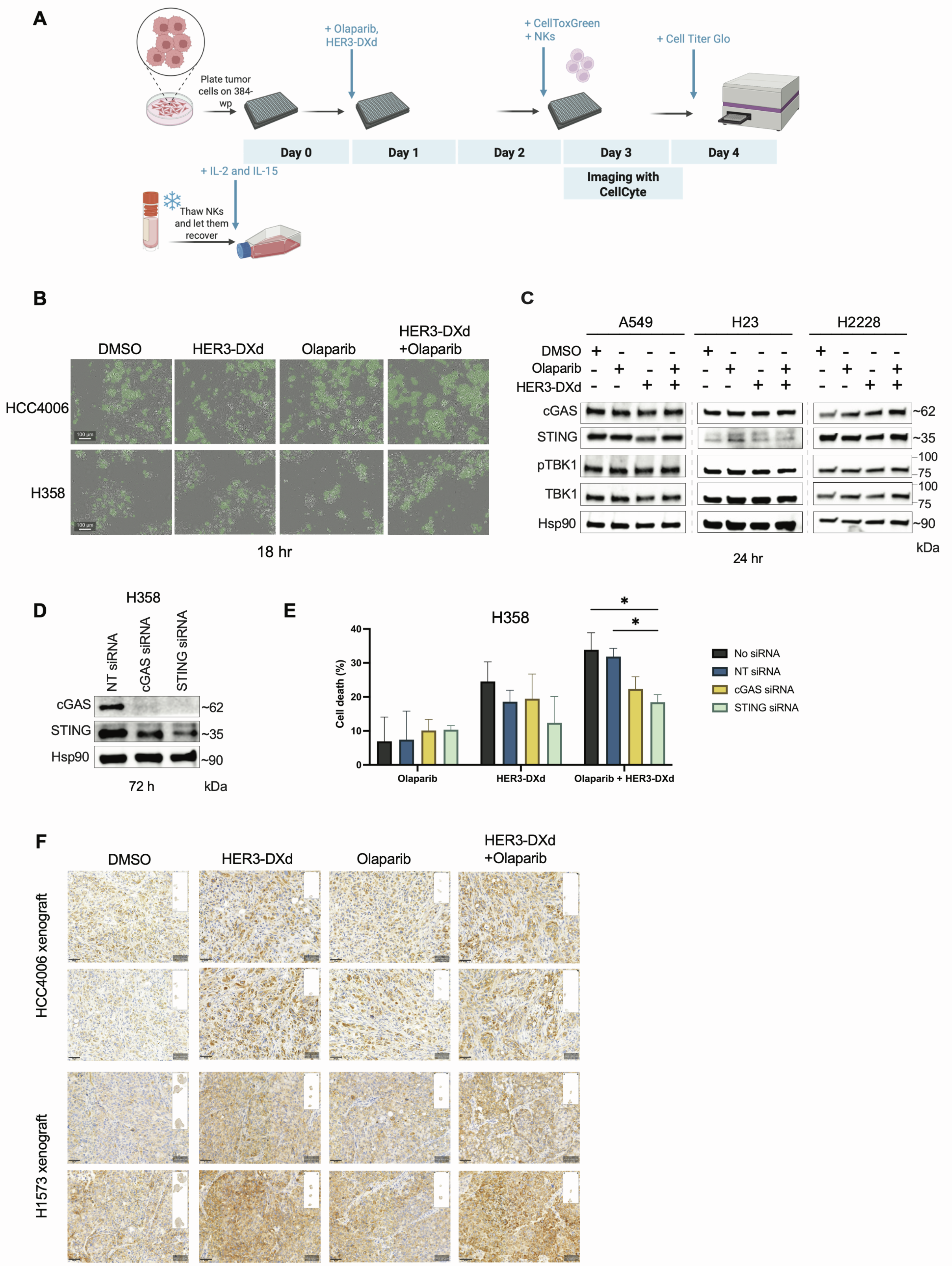

A
+ Olaparib, HER3-DXd
+ CellToxGreen
+ NKs
+ Cell Titer Glo
Plate tumor cells on 384-wp
Day 0
Day 1
Day 2
Day 3
Day 4
Imaging with CellCyte
+ IL-2 and IL-15
Thaw NKs and let them recover
B
DMSO
HER3-DXd
Olaparib
HER3-DXd +Olaparib
HCC4006
H358
18 hr
C
A549
H23
H2228
DMSO
Olaparib
HER3-DXd
cGAS
STING
pTBK1
TBK1
Hsp90
~62
~35
100
75
100
75
~90
kDa
24 hr
D
H358
NT siRNA
cGAS siRNA
STING siRNA
cGAS
STING
Hsp90
~62
~35
~90
72 h
kDa
E
H358
Cell death (%)
40
30
20
10
0
Olaparib
HER3-DXd
Olaparib + HER3-DXd
*
*
No siRNA
NT siRNA
cGAS siRNA
STING siRNA
F
DMSO
HER3-DXd
Olaparib
HER3-DXd +Olaparib
HCC4006 xenograft
H1573 xenograft

Figure S5: Cytotoxicity assessment and cGAS–STING pathway activation across NSCLC cell lines. (related to Figure 6). (A) Experimental design for the NK killing assay. (B) Representative images of cytotoxicity visualized with CellCyte live cell imaging and CellTox Green in *EGFR*-mutated HCC4006 and *KRAS*-mutated H358 cells after 18 hours of NK cell addition. Scale bar, 100 µm. (C) Western blot analysis of cGAS-STING pathway activation in A549, H23, and H2228 cells treated with olaparib (1 µM), HER3-DXd (10 µg/mL), or their combination, n = 3 biological replicates. Hsp90 was used as a loading control. (D) Western blot analysis of cGAS and STING expression following siRNA-mediated silencing of cGAS or STING on H358, assessed at 72 h, n = 3 biological replicates. Hsp90 was used as a loading control. (E) NK cell cytotoxicity in H358 cells after siRNA-mediated knockdown of cGAS or STING, treated with olaparib (1 µM), HER3-DXd (10 µg/mL), or their combination. Data are presented as mean ± SD from three biological replicates, with five technical replicates per biological replicate. Two-way ANOVA; *P < 0.05. NT, non-targeting control. (F) Immunohistochemical analysis of cGAS in paraffin-embedded HCC4006 and H1573 xenograft tissues (n = 2 per condition). Treatments included HER3-DXd (3 mg/kg), olaparib (100 mg/kg), or their combination. Scale bar, 50 µm.

Table S1: IC50 values in µM for tested inhibitors (related to Figure 1D).

	HCC4006	H1975	DFCI-243	DFCI-284	PC-9
Olaparib (PARPi)	~ 0.2289	-0.3276	~5.384e+014	0.1875	1,414
MK1775 (WEE1i)	0.2746	-1,074	0.05982	-1,310	0.103
Ceralasertib (ATRi)	1,407	-0.5089	0.4208	-0.3744	0.4342
Prexasertib (CHK1i)	~ 1.832e-017	~ -6.562	0.00093	-3,183	0.00129
Barasertib (AURKBi)	~ 20.99	-1,592	0.00256	-2,516	0.01725
CFI-402257 (MPS1i)	0.1114	-1,495	0.02856	-1,691	0.02919
Volasertib (PLKi)	~ 9.059e+028	~ -16.55	0.00337	-2,986	0.00961
Rucaparib (PARPi)	~ 0.0009312	-3,525	~ 850.5	0.6863	0.7612
MK5108 (AURKAi)	~ 0.1139	-0.9011	0.318	-0.793	0.178
Veliparib (PARPi)	0.0316	~ -2.281	~ 0.003230	~ -3.547	~ 0.2933
KU-55933 (ATMi)	1,384		~ 34177	~ -0.08551	~ 1.333
Nutlin-3a (MDM2i)	~		~ 0.000	-0.3424	~ 0.1747
NSCCHK109555 (CHK2i)	NA		~ 0.004931		
PFTb			~ 0.05238		
